# Ecogeographical Variation in Skull Shape of South-American Canids: Abiotic or Biotic Processes?

**DOI:** 10.1007/s11692-015-9362-3

**Published:** 2015-12-07

**Authors:** Jamile de Moura Bubadué, Nilton Cáceres, Renan dos Santos Carvalho, Carlo Meloro

**Affiliations:** Programa de Pós-Graduação em Biodiversidade Animal, CCNE, Federal University of Santa Maria, Santa Maria, RS 97110-970 Brazil; Department of Ecology and Evolution, CCNE, Federal University of Santa Maria, Santa Maria, RS 97110-970 Brazil; Research Centre in Evolutionary Anthropology and Palaeoecology, School of Natural Sciences and Psychology, Liverpool John Moores University, Byrom Street, Liverpool, L3 3AF UK

**Keywords:** Canidae, Carnivora, Climatic adaptations, Geographic clines, Interspecific competition, Macroecology

## Abstract

Species morphological changes can be mutually influenced by environmental or biotic factors, such as competition. South American canids represent a quite recent radiation of taxa that evolved forms very disparate in phenotype, ecology and behaviour. Today, in the central part of South America there is one dominant large species (the maned wolf, *Chrysocyon brachyurus*) that directly influence sympatric smaller taxa via interspecific killing. Further south, three species of similar sized foxes (*Lycalopex* spp.) share the same habitats. Such unique combination of taxa and geographic distribution makes South American dogs an ideal group to test for the simultaneous impact of climate and competition on phenotypic variation. Using geometric morphometrics, we quantified skull size and shape of 431 specimens belonging to the eight extant South American canid species: *Atelocynus microtis*, *Cerdocyon thous*, *Ch. brachyurus*, *Lycalopex culpaeus*, *L. griseus*, *L. gymnocercus*, *L. vetulus* and *Speothos venaticus*. South American canids are significantly different in both skull size and shape. The hypercarnivorous bush dog is mostly distinct in shape from all the other taxa while a degree of overlap in shape—but not size—occurs between species of the genus *Lycalopex*. Both climate and competition impacts interspecific morphological variation. We identified climatic adaptations as the main driving force of diversification for the South American canids. Competition has a lower degree of impact on their skull morphology although it might have played a role in the past, when canid community was richer in morphotypes.

**Electronic supplementary material:**

The online version of this article (doi:10.1007/s11692-015-9362-3) contains supplementary material, which is available to authorized users.

## Introduction

Understanding species community assembly is one of the central aims of macroecology (Rosenzweig 1995). For instance, on the one side, we expect abiotic forces, such as climate and geographical barriers, to apply filters influencing species distribution and community composition. On the other side, biotic competition might produce unexpectedly stable species assemblages. Such balancing forces are clearly not mutually exclusive and we have strong evidence that the majority of animal groups tend to be quite resilient and less sensitive to abiotic forces than expected by theory (Vrba 1993). In this regards, members of the mammalian order Carnivora received particular attention for being endothermic, ecologically diverse and secondary consumers (Goswami 2010). The red fox (*Vulpes vulpes*), for instance, is the commonest species to provide evidence for the impact of climate on phenotypes, particularly in skull size, which varies with latitude (Churcher 1960; Davis 1977; Cavallini 1995; Yom-Tov and Geffen 2006; Meiri et al. 2007; Yom-Tov et al. 2007; Szuma 2008). Other lines of investigations on carnivorans’ skull took direct competition into account for explaining carnivoran assembly rules (Dayan et al. 1989, 1992; Dayan and Simberloff 2005; Meiri et al. 2011).

Carnivora generally exhibit broad range of ecological and phenotypic variation accompanied by behavioural attributes (e.g. predatory/killing behaviour) that makes them particularly sensible to biotic processes (Palomares and Caro 1999; Donadio and Buskirk 2006). Here, we use South-American canids as model species to test the hypothesis that climate and competition might have a direct impact on species morphological variation at broad geographical scale. South-American canids represent a recent radiation due to their late Pliocene colonization from a restricted pool of North American taxa (Berta 1987; Prevosti 2010; Perini et al. 2010; Wang et al. 2008). Despite this, South America holds more than 10 living canid species, being the largest extant regional diversity found in the world (Prevosti et al. 2009a; Perini et al. 2010; Sillero-Zubiri et al. 2004). Its endemic taxa includes species with a broad diversity of body size and feeding ecology such as the large (average mass = 25 kg) omnivorous maned wolf *Chrysocyon brachyurus* (Illiger, 1815) and the small (mass ranging 5–8 kg) hypercarnivorous bush dog *Speothos venaticus* (Lund, 1842) (Sillero-Zubiri et al. 2004).

Diversity of fox-like ecomorphs is also broad including species such as the widely distributed crab-eating fox (*Cerdocyon thous*) and the four species of *Lycalopex* that share to some extent geographic range, especially in Argentina, and omnivorous feeding habits. Such a high diversity was even higher during the prehistory (Prevosti 2010; Perini et al. 2010) with larger carnivorous wolflike forms possibly influencing ecology and distribution of smaller taxa.

Currently, we have direct evidence on the impact of competition on ecology and behaviour of extant South-American canids. Di Bitetti et al. (2009) recorded patterns of behavioural shift by *Lycalopex gymnocercus* due to competition with *C. thous*. In sympatry, *L. gymnocercus* changes its normal activity to prevent confrontation with the larger *C. thous*. Also, interspecific killing occurs quite intensively, with large taxa, such as *Ch. brachyurus*, generally controlling densities and behaviour of smaller species (Donadio and Buskirk 2006; Oliveira and Pereira 2014).

Skulls of South-American wild dogs received reasonable attention to clarify patterns of growth and taxonomy (Segura and Prevosti 2012; Segura 2013), paleobiological and ecological adaptations (Prevosti et al. 2005; Prevosti et al. 2009a, b), and more recently climatic impact (Machado and Hingst-Zaher 2009; Martinez et al. 2013), but little is known about the impact of competition on their intra and interspecific variation. Wayne et al. (1989) identified high degree of morphological divergence between South-American foxes in spite of their relatively short time of divergence (<250,000 years) and we might expect this to occur even strongly when larger taxa are included.

By focusing our investigation on both climate and competition, we aim to provide a fully comprehensive framework to interpret skull morphology of the extant South-American canids at broad, continental scale. We opted to quantify skull size and shape by using geometric morphometrics as a good proxy for phenotypic variation at broad geographical scale (see also Cáceres et al. 2014; Meloro et al. 2014a, b). This method was favoured among others because it allows direct and independent visualizations of size and shape patterns together with higher statistical power with reasonably large sample sizes (Adams et al. 2004, 2013).

More specifically our aim is to test the following hypotheses:

1. South American canids differ in both skull size and shape;
2. Size influences shape differences between species;
3. Skull shape and size co-vary with climatic variables and degree of competition across species;
4. Species follow distinct phenotypic patterns of skull shape changes in relation to broad environmental variables or to different degrees of competition.

Hypothesis 1 concerns the biological paradigm of interspecific differentiation and functional convergence. Functional convergence in the skull of carnivorans has been very often detected in relation to extreme feeding adaptations (e.g. durophagy, Figueirido et al. 2011, 2013 or solitary hunting, Meloro et al. 2015a). Meloro (2011), Meloro and Raia (2010) and Meloro and O’Higgins (2011) identified morphological similarities also between omnivores (i.e., canids and viverrids) and we might expect some overlap between South-American taxa with similar diet. Hypothesis 2 relates to the recent findings by Cáceres et al. (2014) and Meloro et al. (2014a, b) on geographical variation of capuchin and howler monkeys: in these cases a significant allometric component was detected also across geographic localities so that skull shape differences were mostly influenced by size.

Hypotheses 3 and 4 relate to ecogeographical pattern generally identifiable in the mammalian skull (Cardini et al. 2007). If abiotic forces are more relevant to regulate canid community assembly we might expect stronger co-variation between skull morphology and climate then between skull morphology and competition. The opposite might occur if biotic forces are more relevant.

## Materials and Methods

### Raw Data and Geometric Morphometrics

**Fig. 1:**
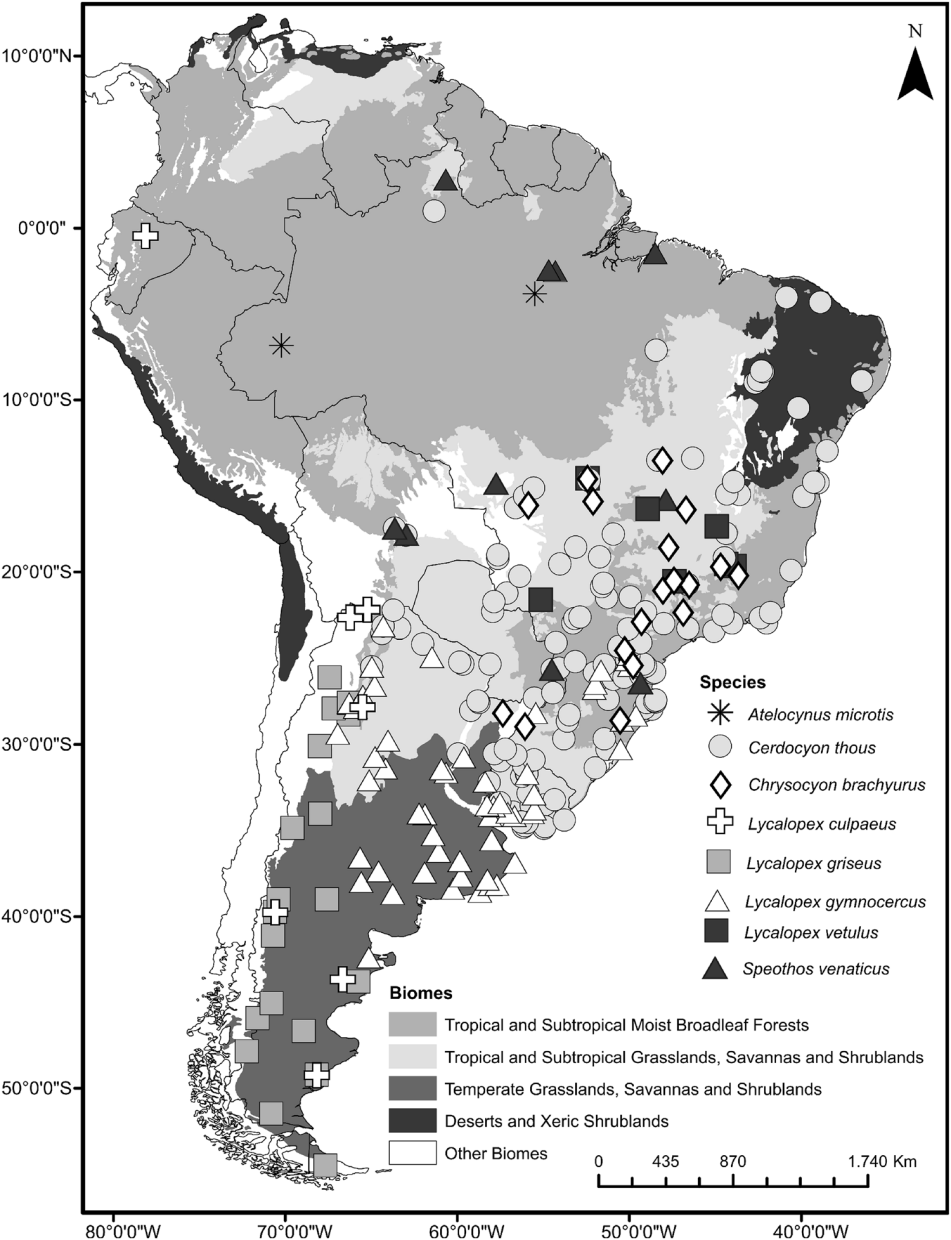
Map of South America showing the geographic distribution of canid skull specimens. Sampling localities of different species are shown by *different symbols*

Our sample includes skull pictures in ventral view of 431 wild-caught adult canid specimens of South America (Online Resource). For each specimen we recorded the geographic coordinates of its collection locality resulting in 262 different localities covering seven countries (Fig. 1; see also Table 1).

**Table 1:** Skull sample size for the eight species of canids included in this study

Species	# Specimens	# Females	# Males	Undet
*Atelocynus microtis* (Sclater, 1882)	4	1	3	0
*Cerdocyon thous* (Linnaeus, 1766)	227	71	94	62
*Chrysocyon brachyurus* (Illiger, 1815)	25	5	7	13
*Lycalopex culpaeus* (Molina, 1782)	13	5	4	4
*Lycalopex griseus* (Gray, 1837)	32	9	4	19
*Lycalopex gymnocercus* (Fischer, 1814)	99	38	28	33
*Lycalopex vetulus* (Lund, 1842)	16	7	5	4
*Speothos venaticus* (Lund, 1842)	15	3	10	2
Total	431	139	155	137

Skull pictures were taken at fixed distance (2 m). This procedure standardizes the sample of digital images and minimizes deformation due to the lenses (as in Meloro et al. 2008). When taking pictures, we set up a scale bar adjacent to the specimen in order to transform digital pixels in linear measurements (Zelditch et al. 2004).

Ventral view was chosen because the palate of canids is relatively flat and the teeth can be individually recognized. Digital photographs were landmarked by one of us (JMB) using the tpsDig2 ver. 2.16 (Rohlf 2015). Landmarking by only one investigator allowed to minimize inter-observer error and repeated sessions on 10 random specimens were taken to assess landmark repeatability (Cardini and Tongiorgi 2003; Meloro 2011; Meloro et al. 2014a).

**Fig. 2:**
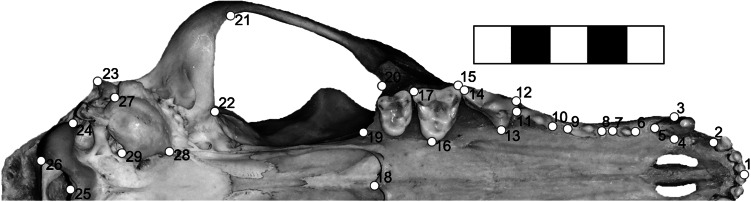
Disposition of 29 landmarks on a skull of *Chrysocyon brachyurus* specimen. *1* Midpoint of central incisors; *2* posterior-most point of lateral incisor alveolus; *3–5* canine area; *6–7* first pre-molar length; *8–9* second pre-molar length; *10–11* third pre-molar length; *12–14* fourth pre-molar (carnassial) area; *15–17* first molar area; *18* most posterior tip of the palatine; *19–22* zygomatic arch area; *23* tip of paracondylar process; *24–26* occipital condyle area; *27–29* auditory bulla area

In order to describe effectively craniodental morphology, we digitized 29 homologous landmarks (Fig. 2). The landmarks recorded the overall skull shape, zygomatic arch, rostrum (palate), auditory bulla, and position and size of the teeth, being all features generally related to feeding adaptations in Carnivora and Canidae (Schutz et al. 2009; Segura and Prevosti 2012; Meloro et al. 2015b).

Generalized procrustes analysis (GPA, Rohlf and Slice 1990) allowed us to remove from the original landmark coordinates differences in size, position and orientation. This procedure transforms raw landmarks coordinates into shape variables: the procrustes coordinates. The GPA coordinates were projected into a weight matrix to characterize shape using non-affine (Partial Warps) and affine (uniform) components of thin plate spline. A principal component analysis of the weight matrix (=relative warp analysis, RWA) allowed us to visualize shape changes of each specimen relative to the mean shape (Zelditch et al. 2004). Skull size was directly extrapolated from raw landmark coordinates using the centroid size (Rohlf 2000). We used the software tpsRelw version 1.49 (Rohlf 2015) to compute GPA, extract the weight matrix, and to compute a RWA.

### Statistical Analyses

#### Taxonomy and Sexual Dimorphism

To prevent pseudoreplication and have better estimation of shape and size in each locality, we used the average values of our geometric morphometric results per locality and sex in all statistical analyses (Cardini et al. 2007; Cáceres et al. 2014).

We used ANOVA and MANOVA to test if canid species, used as factor named “taxonomy”, differ in skull size and shape. Sex was also included as factor in the models where taxonomy and sex were tested for interaction (Cardini and Elton 2008). Scores of a selection of Relative Warp axes that explained at least 95 % of variance were included as dependent variables in the MANOVA models to reduce degrees of freedom in relation to sample size of particularly small groups (Meloro and O’Higgins 2011).

#### Allometry

In order to quantify the allometric effects on our sample, we used the natural log transformed centroid size (=lnCS) in all the analyses as an independent variable. The strength of allometric signal was tested separately in the whole (N = 461) and in the locality averaged sample (N = 262) of skulls using MorphoJ (Klingenberg 2011) by performing a multivariate regression of lnCS on skull shape (cf. Cáceres et al. 2014; Meloro et al. 2014a, b). Together with global allometry (that assumes no difference in slope occurs between species subgroups) we also tested for allometry using pooled regression within subgroups (Klingenberg 1996). Using the R environment, version 2.8.1 (R Development Core Team 2013) and the package geomorph (Adams and Otarola-Castillo 2013) we tested for differences in allometric slope between species by running an ANOVA model with interaction using shape (as procrustes distances) as dependent variable, ln CS as covariate and species as factor. We run 9999 permutations to validate reliability of the *P* value. Additionally, the global and locality averaged skull sample were subdivided into single species dataset in order to identify the strength of allometry in different taxa and link these results to taxon-specific variation partitioning models (see below).

#### Ecogeographical Variation

To test for the impact of geography on skull shape we employed each specimen collection locality to extract nineteen bioclimatic variables with a resolution of 10′ from the WorldClim raster database (Hijmans et al. 2005) by using the DIVA-GIS 7.5 software (http://www.diva-gis.org/download).

Two block partial least squares (=PLS; Rohlf and Corti 2000) was applied using tpsPLS v.1.18 (Rohlf 2015) to test the relationship between climate and skull shape. PLS extracts vectors from the correlation matrix of each block so that the degree of co-variation between one block and the other is maximized (Rohlf and Corti 2000). In MorphoJ (Klingenberg 2011) we also employed PLS to test the correlation between size (lnCS) and the 19 bioclimatic variables. Although this procedure generates only one pair of vectors, it allows performing comparisons with PLS vectors obtained for shape (see Meloro and Jones 2012).

#### Competition

To identify the possible impact of competition on skull shape and size we ascribed to each geographic locality the presence/absence of distinct canid species that were characterized according to their diet, body mass, and biome preference (Sillero-Zubiri et al. 2004).

For each focal species recorded at a certain locality we recorded the number of canid taxa that could potentially interact with it due to dietary adaptation, habitat preference and body size. For each locality we recorded the number of canids with the same diet and habitat and standardized these values for the total number of species potentially present. Index for assessing the potential of interspecific killing (here named body size factor) was computed through Donadio and Buskirk (2006) arcsine square root of BSC defined as the body size difference for each species pair, which is calculated through the equation BSD = (*Mb*_*l*_ − *Mb*_*s*_)/*Mb*_*l*_, where *Mb*_*l*_ is the mass of the larger species and *Mb*_*s*_ the mass of the smaller one. If the result of arcsine $\sqrt {BSD}$ was between 2 and 5.4, than we could consider the pair of species as potential competitors directly influencing each other via interspecific killing (see Donadio and Buskirk 2006 for more details). For example, at locality X we recorded a skull of *C. thous*. In this locality the species could potentially interact with *L. gymnocercus* due to habitat preference (open grasslands) and diet overlap (Vieira and Port 2007), but not by interspecific killing because the pair *C. thous* and *L. gymnocercus* has arcsine $\sqrt {BSD}$ lower than two (see Donadio and Buskirk 2006). *Speothos venaticus* is also present at this locality. This last species is not a potential competitor with *C. thous* in any of our factors because this pair of species arcsine $\sqrt {BSD}$ is lower than 2 (see Donadio and Buskirk 2006). They do not overlap in dietary adaptation due to the fact that *C. thous* presents an omnivorous diet while *S. venaticus* is a specialized meat eater. For habitat preference, *C. thous* prefers open grassland areas (Berta 1982) and *S. venaticus* is primarily a forest dweller (Oliveira 2009). Thus, we have a value of 0 for interspecific killing and 0.5 for diet and 0.5 for habitat (half of the species in that location is a potential competitor due to diet and habitat preferences). We performed this calculation for every specimen, constructing a table that was then used to test competition via PLS between shape or size and the competition data matrix.

#### Angular Comparison

After assessing via PLS the impact of climate and competition versus skull shape, MorphoJ 1.05 (Klingenberg 2011) was used to compare the direction of PLS shape vectors extracted for climate and competition, separately. This provides a direct test to assess the impact of these factors on shaping canid communities and phenotypic changes. Such a test was not available for PLS size for which we opted to simply compare strength of correlation between the samples. If climate impacts size more strongly than competition we might expect higher correlation coefficient (=r) in PLS.

#### Variation Partitioning

We employed variation partitioning (Diniz-Filho and Bini 2008; Raia et al. 2010; Meloro et al. 2014a) to evaluate the singular contribution to skull shape variance of four distinct components: taxonomy (described by the categorical variable “species”), size (described by lnCS), climate (described by the nineteen bioclimatic indices) and competition (described by the three variables of size, diet and biome overlap between the species). These factors are all considered as predictors (X) of skull shape (Y, described by the 54 shape variables, 2n − 4 where n is the number of landmarks, Rohlf and Slice 1990) into multiple multivariate regression models. We tested for the effect of each single factor in isolation and in interaction with each other using the R package vegan 2.0 (Oksanen et al. 2012). We also analyzed the contribution of different predictors into size variation. We performed variation partitioning between all the average per localities (N = 262; using taxonomy, climate and competition as factors). These procedures were employed for the overall sample of South-American canids and nested subsets of the genera *Cerdocyon* and *Lycalopex* that got sufficient data to be considered separately. Since *Lycalopex* is the only genus that is not monophyletic, we also included taxonomy in its model. The other genera were not analyzed separately due to low number of localities.

#### Comparative Methods

Skull size and shape values together with bioclimatic and competition parameters were averaged across species in order to identify correlation patterns at macroevolutionary (above species) level.

This new dataset included eight data points only. We tested again for allometric patterns in between species as well as impact of climate and competition using Partial Least Squares. Due to species shared ancestry, we firstly produced a molecular phylogeny of our selected taxa using the 10 k tree project database (Arnold et al. 2010, Online Resource). This database provided access to all updated molecular data of extant Canidae and generated consensus phylogenetic tree based on a baysean approach using selected taxa only. MorphoJ was employed to test for the presence of a phylogenetic signal in our shape data comparing the observed sum of procrustes distances between the eight species averaged shapes and their reconstructed ancestral node values versus the distribution of these sums obtained randomizing tips and node values (cf. Klingenberg and Gidaszewski 2010; Meloro and Jones 2012). Regressions and Partial Least Squares models were eventually repeated on independent contrasts using MorphoJ in order to re-evaluate the influence of allometry, climate and competition on macroevolutionary scale (cf. Meloro et al. 2014a, b).

## Results

### Canids Skull Shape

**Fig. 3:**
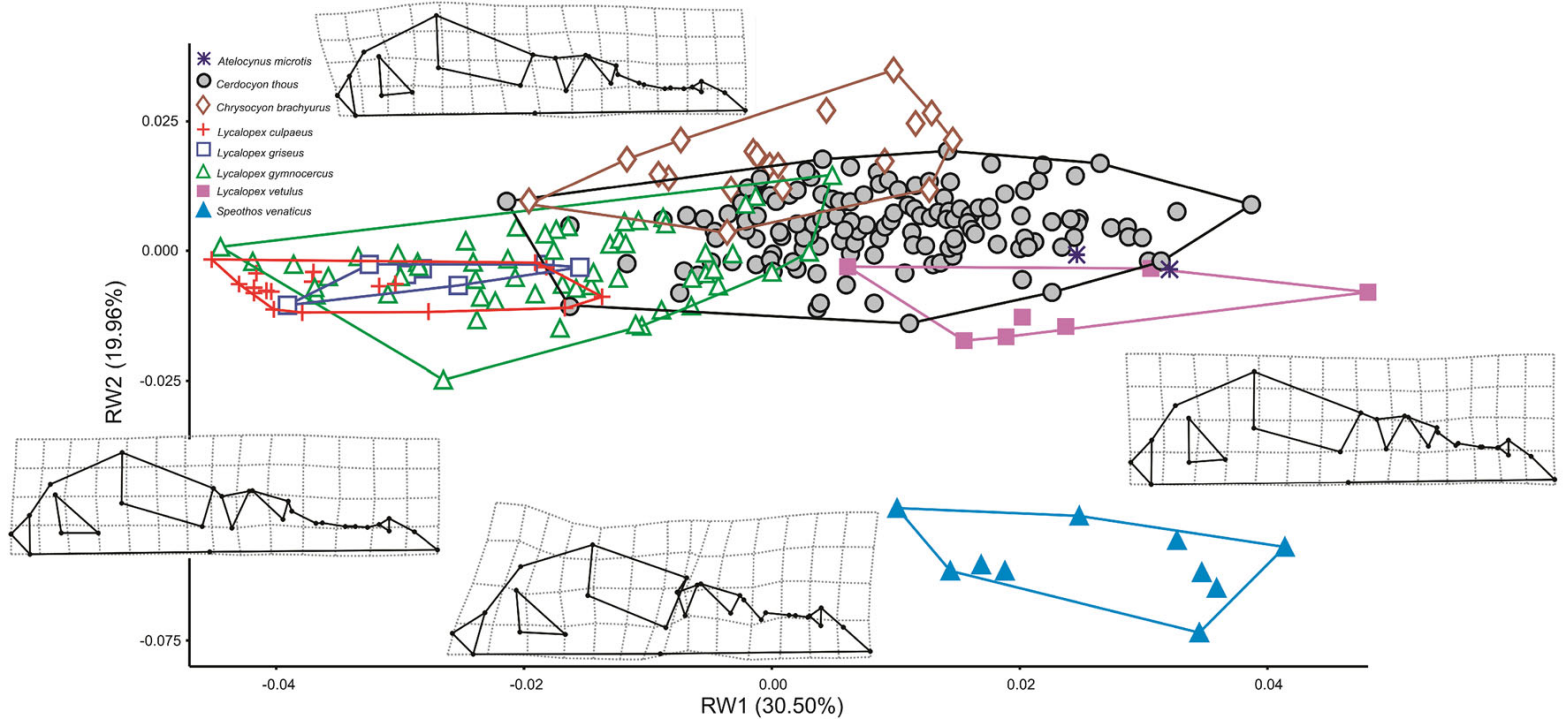
Scatter plot of RW1 versus RW2. Transformation grids visualize shape deformations relative to the mean at the positive and negative extremes of Relative Warps axes. Every species is labeled according to *different color* and *symbol* within minimum convex hull superimposed (Color figure online)

The first twenty five Relative Warps cumulatively explain 95 % of total variance. Plotting the first (30.50 % of shape variance) versus the second (19.96 %) RWs evidence extensive overlap between the different canids’ species, although RW1 separates genera, showing some segregation between almost all *Lycalopex* spp. and the others (Fig. 3). Only *S. venaticus* did not overlap with others species. RW1 describes changes in the zygomatic arch, occipital condyle, auditory bulla, muzzle and teeth. Species at the extreme negative of RW1 exhibit smaller zygomatic arch, occipital condyle and auditory bulla, more elongated and thinner muzzle, larger first molar and carnassial and smaller canine and incisors. The RW2 describes shape changes related to braincase, zygomatic arch, occipital condyle, auditory bulla, muzzle area and teeth row positioning and size. On the negative scores of RW2 specimens have larger braincase, zygomatic arch, occipital condyle and auditory bulla, shorter and thicker muzzle, small first molar and larger carnassial, canine and incisors.

MANOVA performed using the first 25 RWs evidenced significant shape differences between species (N = 262, Pillai’s trace = 4.116; F_175, 1652_ = 13.471; *P* ≪ 0.001). When only locality average sexed individuals were analyzed (N = 206), two-way MANOVA shows no significant differences between sex (Pillai’s trace = 0.173; F_25, 165_ = 1.385; *P* = 0.118), but confirms significant differences between species (Pillai’s trace = 4.146; F_175, 1197_ = 9.936; *P* ≪ 0.001), with no interaction between the two factors (Pillai’s trace = 0.895; F_175, 1197_ = 1.003; *P* = 0.477). Pairwise comparisons using the first two RWs (50.46 % of total variance) revealed that all species differ in skull shape, expect for the pair *Lycalopex culpaeus*–*L. griseus*, *L. culpaeus*–*L. gymnocercus* and *Atelocynus microtis*–*L. vetulus* (Table 2).

**Table 2:** Pairwise comparisons between South-American canid species for skull shape

	*A. microtis*	*C. thous*	*Ch. brachyurus*	*L. culpaeus*	*L. griseus*	*L. gymnocercus*	*L. vetulus*	*S. venaticus*
*A. microtis*		**0.008**	≪**0.001**	**< 0.001**	≪**0.001**	≪**0.001**	0.275	≪**0.001**
*C. thous*	4.924		≪**0.001**	≪**0.001**	≪**0.001**	≪**0.001**	≪**0.001**	≪**0.001**
*Ch. brachyurus*	29.160	45.111		≪**0.001**	≪**0.001**	≪**0.001**	≪**0.001**	≪**0.001**
*L. culpaeus*	40.531	41.745	34.926		0.107	0.142	≪**0.001**	≪**0.001**
*L. griseus*	38.199	150.730	115.400	2.472		≪**0.001**	≪**0.001**	≪**0.001**
*L. gymnocercus*	15.378	126.780	63.463	2.0208	14.211		≪**0.001**	≪**0.001**
*L. vetulus*	1.6110	28.661	84.289	43.912	67.453	48.654		≪**0.001**
*S. venaticus*	67.480	496.61	447.590	205.290	410.350	390.020	107.510	

Upper diagonal corresponds to *P* values and lower diagonal corresponds to F values. Significant is highlighted

### Skull Size

**Fig. 4:**
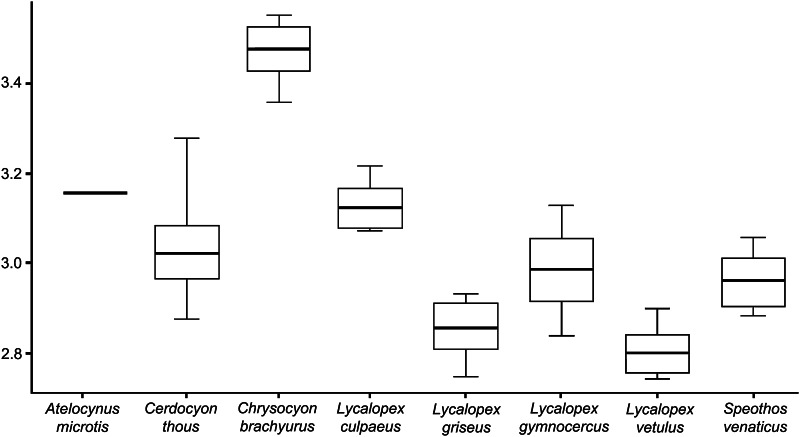
*Box plot* with standardized deviation of natural log transformed centroid size (lnCS) across the South-American canid species. *Black string* median, *white box* first interquartile, *bar* second interquartile

Two-way ANOVA (N = 206) revealed canid species to be significantly different in skull size (F = 123.069, *df* = 7, *P* ≪ 0.001). Males and females also differ in skull size (F = 7.946, t = 1, *P* = 0.005) but no interaction occurs between taxonomy and sex in skull size (F = 1.057, *df* = 7, *P* = 0.393). Therefore, it was possible to use in subsequent analyses the localities averages including those individuals who lacked sex information. One-way ANOVA confirmed species to be significantly different in size after averaging by geographic localities (F = 194.9, *df* = 7, *P* ≪ 0.001). Paired comparisons revealed that all species differ in size, except the pair *A. microtis*–*L. culpaeus* and *L. gymnocercus*–*S. venaticus* (Table 3; Fig. 4).

**Table 3:** Pairwise *t* test between South-American canid species for skull size

	*A. microtis*	*C. thous*	*Ch. brachyurus*	*L. culpaeus*	*L. griseus*	*L. gymnocercus*	*S. venaticus*
*C. thous*	**0.002**						
*Ch. brachyurus*	≪**0.001**	≪**0.001**					
*L. culpaeus*	0.488	≪**0.001**	≪**0.001**				
*L. griseus*	≪**0.001**	≪**0.001**	≪**0.001**	≪**0.001**	≪**0.001**		
*L. gymnocercus*	≪**0.001**	≪**0.001**	≪**0.001**	≪**0.001**	≪**0.001**		
*S. venaticus*	≪**0.001**	≪**0.001**	≪**0.001**	≪**0.001**	≪**0.001**	0.140	
*L. vetulus*	≪**0.001**	≪**0.001**	≪**0.001**	≪**0.001**	**0.043**	≪**0.001**	≪**0.001**

Significant *P* value is highlighted

### Skull Allometry

Size had a significant impact on skull shape explaining 7.84 % of variance in the global skull sample (N = 431, *P* ≪ 0.0001, see also Online Resource) and 9.46 % of variance in the locality averaged sub-sample (N = 262, *P* ≪ 0.0001, see also Online Resource). ANCOVA models with permutation demonstrated in all cases that slope differ between species (N = 431, F = 8.713, *P* < 0.001; N = 262, F = 1.872, *P* < 0.001). Allometric models using pooled regression within subgroups are also significant in all cases (N = 431, 3.29 Var%, *P* < 0.001; N = 262, 3.77 Var%, *P* < 0.001).

Regression models performed independently for each taxon (Table 4) demonstrates that allometric effect impact species differently. In the bush dog skull size explains the highest percentage of shape variance when compared to the larger *Chrysocyon* and *Cerdocyon*. Within *Lycalopex* allometric shape changes occurs in *L. gymnocercus* only while they are not statistically detectable in the other taxa (see also Online Resource). However, if all *Lycalopex* species are merged in the same sample, skull size explains almost 9 % of shape variance in both complete and locality averaged datasets (Table 4; see also Online Resource).

**Table 4:** Regressions between skull shape and lnCS in different subsamples of canid taxa

Species	Whole sample			Locality averaged		
	N	Var%	*P*	N	Var%	*P*
*Cerdocyon thous*	226	**3.85**	**<0.0001**	143	**4.08**	**<0.0001**
*Chrysocyon brachyurus*	25	**9.28**	**0.0076**	19	**11.52**	**0.0159**
*Lycalopex culpaeus*	13	10.85	0.835	7	25.85	0.0731
*Lycalopex griseus*	33	4.86	0.1003	19	7.19	0.1881
*Lycalopex gymnocercus*	99	**4.20**	**<0.0001**	55	**5.69**	**0.002**
*Lycalopex vetulus*	16	6.68	0.4329	7	26.71	0.0865
*Lycalopex* spp.	161	**9.13**	**<0.0001**	88	**9.02**	**<0.0001**
*Speothos venaticus*	15	**13.44**	**0.0346**	10	**20.81**	**0.0084**

Significance is highlighted

### PLS Climate

**Fig. 5:**
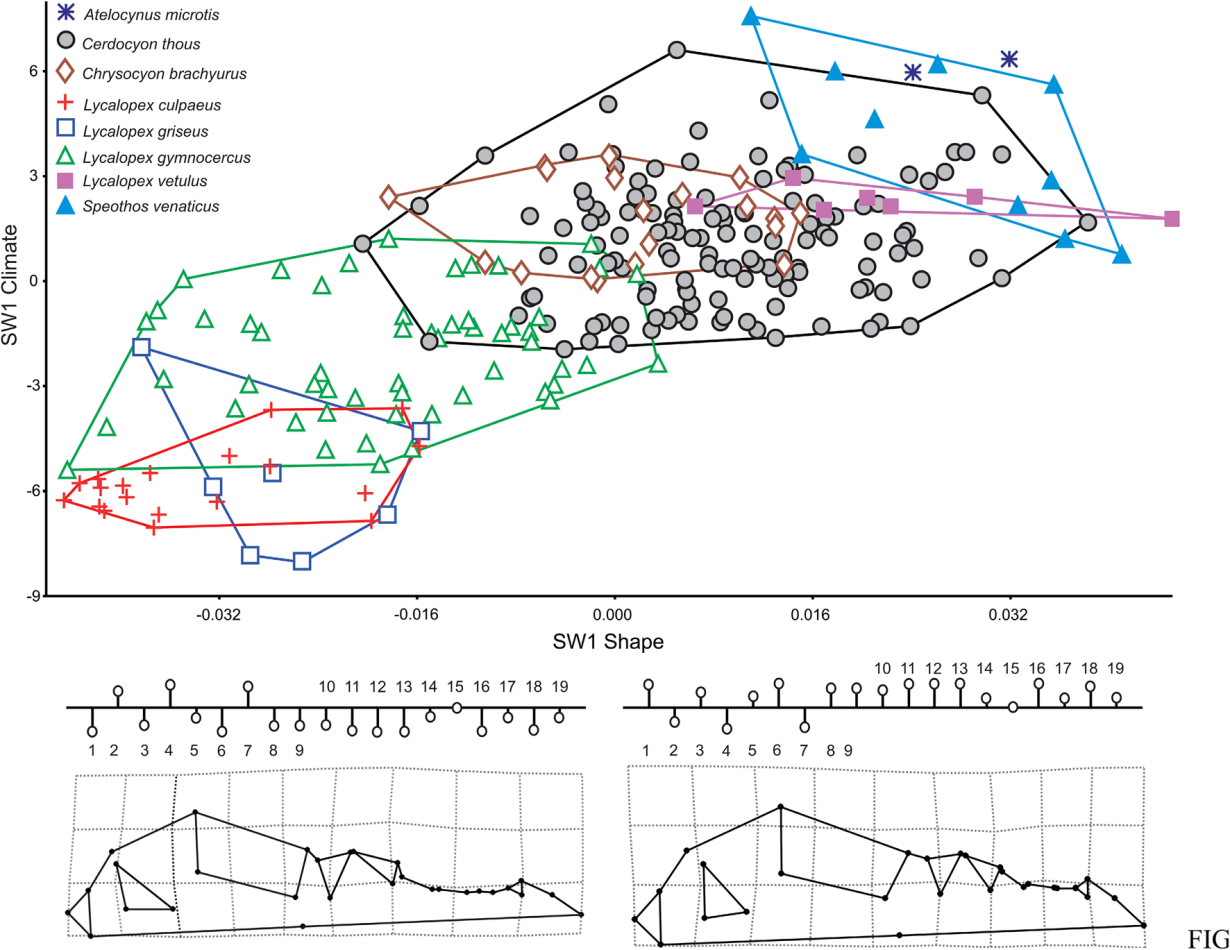
Plot of the first pair of Singular Warps (axis *X* is block shape, axis *Y* is block climate). Below deformation grids and variables profile from the most negative to the most positive Singular Warps scores. Every species is labeled according to *different color* and *symbol* within minimum convex hull superimposed (Color figure online)

Two block Partial Least Squares between 19 bioclimatic variables and skull shape extracts 19 pair of vectors of which the first explains 98.33 % of covariation between the blocks. Correlation between the first pair of axes is strong (r = 0.720) and significant (*P* < 0.001). A scatter plot with Singular Warp 1 of the shape block (SW1 shape) versus SW1 of the climate variables block supports a separation among species along an environmental gradient (Fig. 5). SW1 climate is loaded negatively on Temperature Seasonality (standard deviation × 100) (BIO4, r = −0.864) and Temperature Annual Range (BIO7 = BIO5–BIO6; r = −0.762), while strong positive correlation of SW1 climate scores occurred with Annual Mean Temperature (BIO1, r = 0.864), Min Temperature of Coldest Month (BIO6, r = 0.943), Mean Temperature of Driest Quarter (BIO9, r = 0.794), Mean Temperature of Coldest Quarter (BIO11, r = 0.931), Annual Precipitation (BIO12, r = 0.809), Precipitation of Wettest Month (BIO13, r = 0.891), Precipitation of Wettest Quarter (BIO16, r = 0.896). Therefore, SW1 climate discriminated between seasonal and arid climate (negative scores) versus warm and humid (positive scores).

Species distributed in localities with low precipitation, low mean temperatures and high seasonality exhibited skulls with elongated muzzles, smaller zygomatic arch, larger teeth, narrow auditory bulla and smaller occipital condyle (e.g. *L. griseus*, *L. culpaeus* and most of *L. gymnocercus* representatives). Conversely, *A. microtis*, *S. venaticus*, *L. vetulus*, *Ch. brachyurus* and most of *C. thous* representatives showed positive scores in vector SW1 climate (high precipitation and mean temperature and low seasonality) and therefore had antagonistic skull shape (Fig. 5).

**Fig. 6:**
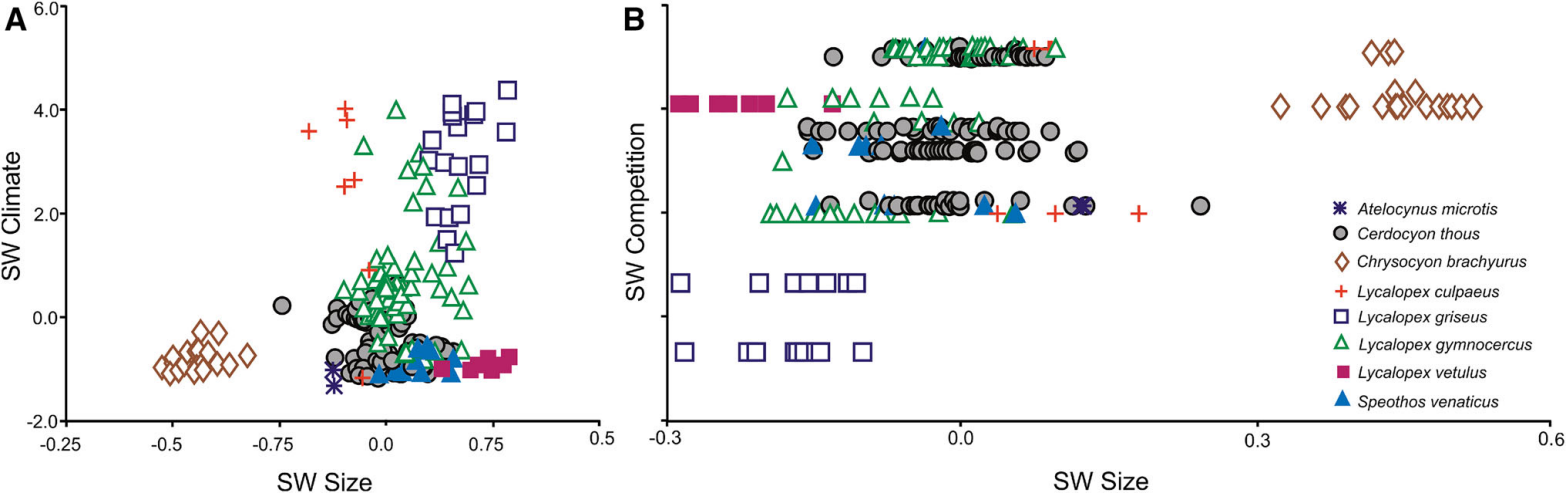
Plot of the pair of Singular Warps. **a** Pls climate plot (axis *X* is block size, axis *Y* is block climate). **b** Plot competiton plot (axis *X* is block size, axis *Y* is block competition). Every species is labeled according to *different color* and *symbol* (Color figure online)

The PLS of size versus climatic variables also extracted a significant pair of axes whose correlation is not particularly strong (r = 0.275; *P* < 0.001). SW climate is loaded positively with Temperature Seasonality (standard deviation × 100) (BIO4, r = 0.827). All *Lycalopex* species occur in high seasonal environments and are characterized by relatively small size (Fig. 6a).

### PLS Competition

**Fig. 7:**
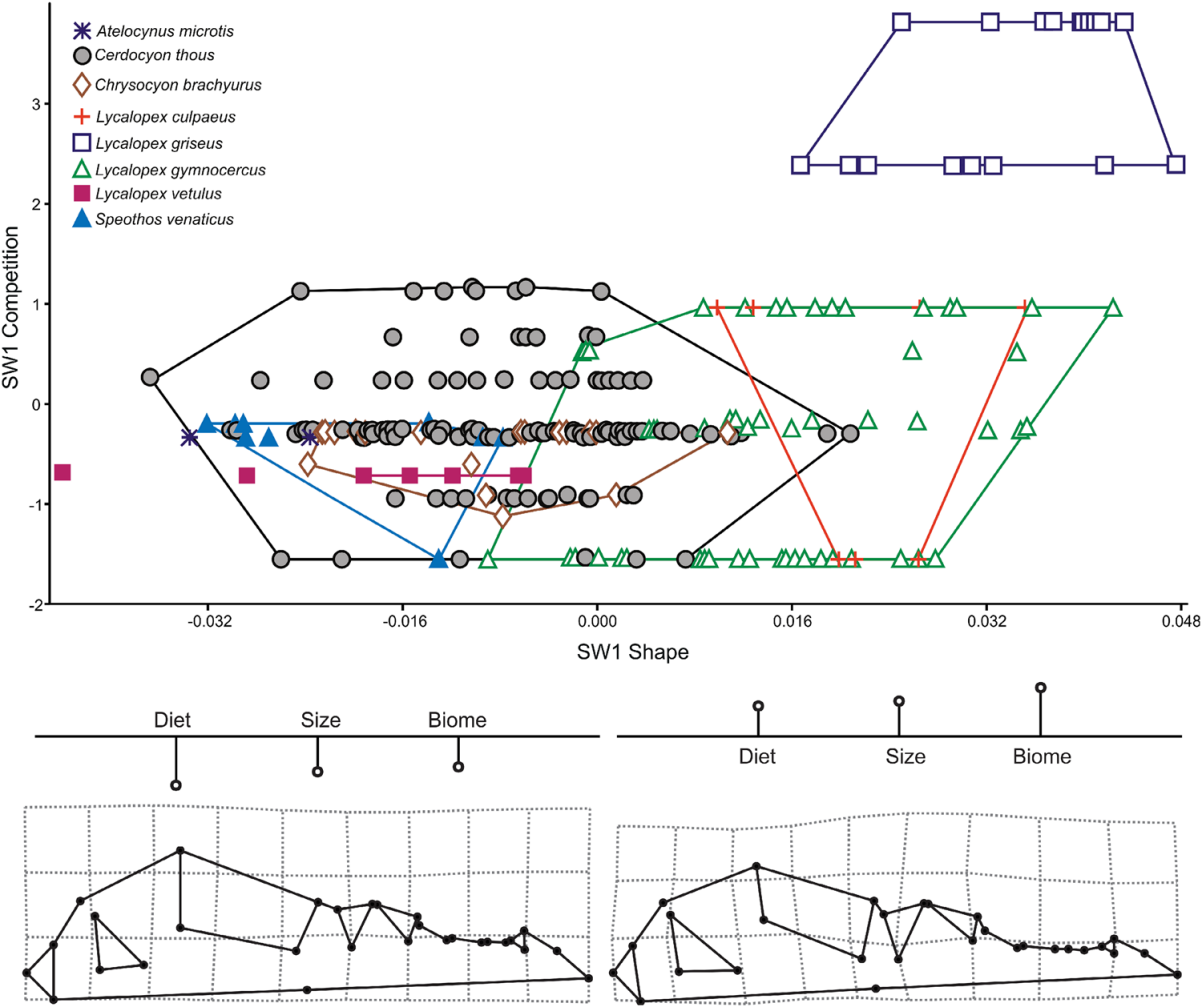
Plot of the first pair of Singular Warps (axis *X* is block shape, axis *Y* is block competition). Below deformation grids and variables profile from the most negative to the most positive Singular Warps scores. Every species is labeled according to *different color* within minimum convex hull superimposed (Color figure online)

Two block Partial Least Squares between competition variables (Diet, Size and Biome) and skull shape extracts three pairs of vectors of which the first explains 70.97 % of covariation between the blocks. Competition has significant impact on skull shape (PLS1, r = 0.419, *P* ≪ 0.001). The scatter plot with Singular Warp 1 of the shape block versus SW1 of competition variables block shows two groups of species that differ among each other the most: the northern group, with *A. microtis*, most specimens of *C. thous*, *Ch. brachyurus*, *L. vetulus* and *Speothos* and the southern group, with mainly *L. griseus* and *L. culpaeus*. *Lycalopex gymnocercus* is right in the middle, overlapping in shape with both groups (Fig. 7). SW1 competition is loaded positively with all three variables (Diet r = 0.823, Interspecific killing r = 0.578 and habitat r = 0.572). Adaptations in the positive end (*L. griseus*, *L. culpaeus* and *L. gymnocercus*) are related to skulls with a larger bulla, a small temporalis insertion area and relatively larger teeth. On negative scores we find specimens of *C. thous*, *Ch. brachyurus*, *L. vetulus* and *S. venaticus.* These specimens show relatively smaller teeth, bulla and larger muzzle.

Significant association also occurs between size and competition (r = 0.332; *P* < 0.001). SW competition is loaded negatively on diet (r = −0.662), size (r = −0.036) and biome (r = −0.748). Species with higher impact of competition are smaller (*L. griseus*) and with less impact of competition are larger (*Ch. brachyurus*) (Fig. 6b).

### Angular Comparison

The direction of PLS shape vectors due to climate and competition are significantly distinct from 90° thus supporting similarities in patterns of skull shape covariation (angle = 18.547°; *P* < 0.001).

Pairwise genera angular comparison for PLS climate revealed that only two pairs of species seem to exhibit similar vector directionality: *Chrysocyon* and *Lycalopex*, and *Cerdocyon* and *Speothos* (Table 5A). As to competition, only *Cerdocyon* and *Lycalopex* exhibit an angle significantly smaller than 90 degrees (Table 5B).

**Table 5:** Pairwise Angular comparisons of SW1 shape vectors of climate (A) and competition (B) between South-American canid genera

	*Cerdocyon*	*Chrysocyon*	*Lycalopex*	*Speothos*
A: Climate				
*Cerdocyon*		0.576	0.329	**0.001**
*Chrysocyon*	85.584		**<0.001**	0.365
*Lycalopex*	82.293	60.375		0.056
*Speothos*	64.420	82.845	74.994	
B: Competition				
*Cerdocyon*	–	0.628	**0.013**	0.651
*Chrysocyon*	86.171	–	0.242	0.449
*Lycalopex*	70.590	80.763	–	0.314
*Speothos*	86.427	84.022	82.048	–

Upper diagonal corresponds to *P* values and lower diagonal corresponds to angles (in °). Significant is highlighted

### Variation Partitioning

**Fig. 8:**
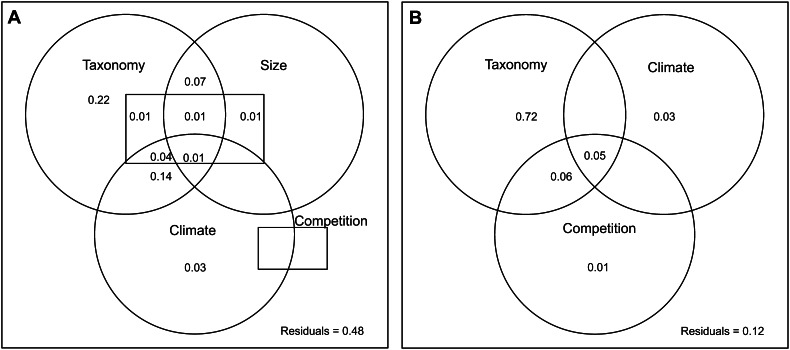
Schematic depiction of the factors analyzed in partition variation meant to illustrate both their individual contribution to shape (**a**) or size (**b**) variance and their interaction components. Values <0 not shown

In the sample of 262 skull shape averages per locality, taxonomy occurs as the most influential variable to explain interspecific shape variation (Adj R^2^ Taxonomy = 0.49). This pattern remains consistent if factors are considered as single “pure” components (Adj R^2^ Taxonomy “Pure” = 0.22, Fig. 8a). Climate is the second most influential variable when considered together with other variables (Adj R^2^ Climate = 0.21) as well as alone (Adj R^2^ Climate “Pure” = 0.03, Fig. 8a). Size and Competition have no influence on shape when considered alone (Fig. 8a). Strong interaction occurs between taxonomy and climate and taxonomy and size (Fig. 8a; Online Resource).

The same strong influence of taxonomy occurs when size is considered a predictor (Adj R^2^ Taxonomy = 0.84). This pattern remains consistent if factors are considered as single “pure” components (Adj R^2^ Taxonomy “Pure” = 0.72, Fig. 8b). After taxonomy, climate is the most influential factor (Adj R^2^ Climate “Pure” = 0.03), followed by competition (Adj R^2^ Competition “Pure” = 0.01, Fig. 8b). Taxonomy interacts with competition and both together interact strongly with climate (Fig. 8b).

The analyses of nested dataset show distinct partition of variance depending on the genus. In *Cerdocyon* size has no significant influence on skull shape as “pure” component. The impact of climate on skull shape is present and significant (Adj R^2^ Climate “pure” = 0.10) while competition explains very little (Adj R^2^ Competition “pure” = 0.01, and see Online Resource). When *Cerdocyon* skull size is modelled as dependent variable, competition is more influential in isolation (Adj R^2^ Competition “pure” = 0.07) while climate has an impact only due to the interaction with the competition (Adj R^2^ Climate × Competition = 0.04; see also Online Resource).

For *Lycalopex*, taxonomy is the most influential factor in explaining variance for skull shape (Adj R^2^ Taxonomy “pure” = 0.05), followed as pure component by climate (Adj R^2^ = 0.04), competition (Adj R^2^ = 0.02) and size (Adj R^2^ = 0.01). A similar pattern occurs when skull size is considered dependent with taxonomy as pure explaining most of the variance (Adj R^2^ = 0.16), followed by climate (Adj R^2^ = 0.08) and competition (Adj R^2^ = 0.02; see also Online Resource).

### Comparative Analyses

When using the record of averaged skull shape (N = 8), allometry is not a significant factor anymore even if skull size appears to explain a substantial proportion of shape variance (16.46 %; *P* = 0.332). The Partial Least Squares confirms the impact of climatic variables on skull shape as detectable by the first significant pair of vectors (93.27 % of covariation explained, r = 0.957, *P* = 0.0083). Association between skull shape and competition is not statistically detectable with PLS (SW1, r = 0.8655, *P* = 0.1954). The test for phylogenetic signal shows that it does not occur in both skull shape (Tree length = 0.0067, *P* = 0.2063) and size (Tree length = 0.218, *P* = 0.4438) making it unnecessary the use of comparative methods.

## Discussion

Our findings show South-American canids are different in skull shape, with peculiar characters occurring especially in *S.venaticus*: large zygomatic arch, big upper carnassial, canine and incisors and short and thick muzzle are all well-established attributes related to its hypercarnivorous diet (Valkenburgh 1991; Kleiman 1972). The tropical hoary-fox (*L. vetulus*) also shows unique skull shape in comparison to other *Lycalopex* southern species. Features such as large auditory bulla and short and thick muzzle make the hoary-fox more similar in skull shape to *Cerdocyon* and *Atelocynus* than to members of its own genus (Fig. 2). In comparison to the southern South-American *Lycalopex*, the hoary-fox is endemic of central Brazil and termites are a large percentage of its diet (Dalponte 2009). The other *Lycalopex* foxes are omnivorous, opportunistic and restricted to colder environments, so this might explain the unusual grouping in cranial shape of *L. vetulus* with other genera (Sillero-Zubiri et al. 2004; Johnson and Franklin 2011).

Skull size also varies considerably among species with *Ch. brachyurus* showing the largest skull. Size differences also occur among members of *Lycalopex* genus confirming and expanding previous observations by Wayne et al. (1989): size differentiation can be interpreted as a mechanism of niche partitioning that eventually allowed these taxa to fulfil different ecological requirements. As such, the role of interspecific allometry in the overall sample is apparent with size explaining almost 10 % of variance in skull shape (generally comparable to that from previous studies, Figueirido et al. 2011, 2013 on all Carnivora and Meloro et al. 2015b on canids only). The ANCOVA model suggests that allometric shape variation differs between taxa to the extent that more specialised morphologies show greater percentage of variance explained by size rather than generalists one (Table 4). Indeed, the hypercarnivorous bush dog (*S. venaticus*) exhibits a strong allometric component when compared to the other taxa in relation to its biomechanically demanding predatory behaviour (Slater et al. 2009). We also note that allometry is not significant for many *Lycalopex* species except *L. gymnocerus*. This possibly might be the result of a broader taxonomic differentiation that occurred in the whole genus in relation to size but not shape characteristics (see Tables 2, 3). Also localized character displacement in certain members of this genus might have favoured size changes not accompanied by dramatic skull shape changes. Prevosti et al. (2013) supports allometry to occur in a 3D skull shape sample of *L. griseus* and *L. gymnocercus* thus corroborating our general result based on the *Lycalopex* as a coherent taxonomic group.

Clearly allometry is a factor in shape differentiation of South American canids, however the PLS and variation partition analyses support a stronger influence of abiotic factors on skull morphology.

Climate seems to represent a stronger factor than competition in explaining skull shape variation. PLS highlights this and also provides the mechanism to visualize shape and size changes related to these factors. Seasonal environments, such as southern South-America, are occupied by non-specialist canid phenotypes, due to *Lycalopex* dominance. Opposite to that, inside the Amazon we have the co-presence of three distinct morphotypes: *Speothos*, *Atelocynus* and the generalist *Cerdocyon*. The peculiar interaction between these genera occurs only in this biome. Compared to other South-American ecoregions where *S. venaticus* is rarely encountered, its species abundance in the Amazon is higher (Jorge et al. 2013). Meanwhile, *A. microtis* is mainly an Amazonian species whose ecological niche relates strongly with wet environments (e.g. main food source is generally fish, Berta 1986; Sillero-Zubiri et al. 2004). From a macroevolutionary point of view this supports the Amazon as a unique ecosystem hosting an ecologically and morphologically diverse canid community with predominance of carnivorous morphotypes (skulls characterised by large zygomatic arch and short rostrum, Fig. 5). We note still all over South America a general pattern of co-presence involving rather three species in different climatic conditions: far from the equator, the co-existence of fox-like phenotypes increases, suggesting that carnivorous phenotypes went extinct and never replaced; in the savannah-Cerrado environments we record skulls of *Chrysocyon*, *Cerdocyon* and *Lycalopex* (depending on the latitude co-occurrence is with *L. gymnocercus* or *L. vetulus*, Fig. 5). Jácomo et al. (2004) report low degree of ecological overlap between *Chrysocyon*, *Cerdocyon* and *L. vetulus* in Cerrado due to differences in feeding habits and activity patterns. The bush dog is also expected to be part of this guild although no data about its diet in co-existence with the other dogs are available. Zuercher et al. (2005) report high consumption of agouti and paca by *Speothos* in Atlantic Forest of eastern Paraguay. If diet of *Speothos* is consistent across the continent we might expect some possible overlap with the maned wolf and the crab-eating fox, however no report of bush dog killing by larger canids occurs in the literature (Oliveira and Pereira 2014).

The size pattern related to climate is not as strong as with shape and we note generally smaller species in the southern part of South America co-existing together (Fig. 6a). The lack of large carnivorous morphotypes is a relatively recent event in the history of southern South-American canids thus explaining it. Fossil record supports this, since *Theriodictis* and *Protocyon* genera (large hypercarnivores) were recorded in southern South America and went extinct very recently at the end of the Pleistocene (Prevosti et al. 2009b).

The co-presence of three distinct morphotypes in size and shape definitely occurs along an environmental gradient and can be better interpreted after looking at PLS of skull morphology versus competition (Fig. 7). All *Lycalopex* foxes exhibit similar shape morphology and high scores being generally the smallest in the canid guild and showing strong overlap with each other across their range. This co-existence seems interestingly favoured by size differences more than shape (Figs. 3, 4) confirming earlier investigation (Fuentes and Jaksic 1979).

The parallelism observed in the PLS vectors suggests that co-variation between shape with climate and competition gradient are two sides of the same coin. Indeed, canid assemblage rules are definitely controlled by environment and South-American species are no exception (Johnson et al. 1996). The largest canid assemblage ever studied is from central Africa with three species of jackals co-occurring together plus hypercarnivore *Lycaon* and the termite specialist *Otocyon*.

Theoretically the Cerrado (a savannah-like vegetation) could support a similar combination of species and we note again similar niche partitioning with two foxes (*Cerdocyon* and *Lycalopex*) that overlap slightly in skull shape but not in size and feeding habits, then the small hypercarnivore bush dog and the large maned wolf.

Variation partitioning provides strong support for abiotic forces as responsible of interspecific morphological differences in South-American canids as a whole but also in the nested dataset of *Cerdocyon* and *Lycalopex* (Fig. 8; Online Resource). Climate consistently explains 3 % of both size and shape variance in isolation, while competition only 1 % for size and none for shape.

In *Cerdocyon* the impact of climate on cranial shape is even stronger (10 %) although it becomes non-significant to explain skull size variation. It can be argued that size changes in *Cerdocyon* are strongly influenced by co-occurrence with larger taxa that might suppress its spectrum of ecological adaptations via direct killing (Oliveira and Pereira 2014). For *Lycalopex* climate also appears to have stronger influence on skull size (8 %) and shape (4 %) than competition.

Overall, these results are a bit counterintuitive considering the rapid adaptive invasion of canids in South America (Perini et al. 2010). This explains the lack of phylogenetic signal encountered in macroevolutionary analyses as well as lack of allometry, but still significant influence of climate. Benton (2009) suggests that competition might regulate animal communities on short time scale, while abiotic forces dominate on big temporal scale. We argue that South-American canids show a mix of both phenomena since prehistoric diversity was higher, when competition could have been more relevant than now (Wang et al. 2008). By adapting flexible ecological feeding niches (with the possible exception of hypercarnivorous *Speothos*) South-American canids might have escaped constrains imposed by competition evolving a flexible morphology.

## Electronic supplementary material

Below is the link to the electronic supplementary material.

Supplementary material 1 (PDF 1306 kb)
