# Supplementary material for: Ecogeographical Variation in Skull Shape of South-American Canids: Abiotic or Biotic Processes?
Source: Evol Biol. 2015 Dec 7;43:145–59. doi: 10.1007/s11692-015-9362-3 (PMC4860408; doi:10.1007/s11692-015-9362-3)
Supplement: Supplementary file 1 — Supplementary material 1 (PDF 1306 kb) [file 11692_2015_9362_MOESM1_ESM.pdf]

EVOLUTIONARY BIOLOGY
Online Resource

Ecogeographical variation in skull shape of South-American canids: abiotic or biotic processes?

Jamile de Moura Bubadué, Nilton Cáceres, Renan dos Santos Carvalho, Carlo Meloro*

***Correspondence**: Carlo Meloro, Research Centre in Evolutionary Anthropology and Palaeoecology, School of Natural Sciences and Psychology, Liverpool John Moores University, Byrom Street, L3 3AF, Liverpool, UK. E-mail: C.Meloro@ljmu.ac.uk

List of 431 canid specimens used for morphometric analyses, with data on species, sex (F, female; M, male; or unknown), museum number, and geographical coordinates.

Species	Sex	Museum number	Latitude	Longitude
Atelocynus microtis	F	MZUSP 4320	-6.84	-70.24
Atelocynus microtis	M	MZUSP 19753	-3.83	-55.49
Atelocynus microtis	M	MZUSP 19754	-3.83	-55.49
Atelocynus microtis	M	MZUSP 19751	-3.83	-55.49
Cerdocyon thous	F	MACN 14322	-25.49	-64.97
Cerdocyon thous	M	MACN 47402	-23.56	-64.40
Cerdocyon thous	M	MACN 47189	-23.14	-64.32
Cerdocyon thous	F	MACN 30345	-23.14	-64.32
Cerdocyon thous	F	MACN 47190	-23.14	-64.32
Cerdocyon thous	M	MACN 486	-23.14	-64.32
Cerdocyon thous	F	MACN 485	-23.14	-64.32
Cerdocyon thous	M	MACN 4810	-23.14	-64.32
Cerdocyon thous	M	MACN 33344	-22.24	-63.73
Cerdocyon thous	F	MACN 5060	-17.46	-63.66
Cerdocyon thous	F	MACN 5061	-17.46	-63.66
Cerdocyon thous	M	MACN 30481	-23.25	-63.35
Cerdocyon thous	F	MN 5497	-17.87	-63.00
Cerdocyon thous	M	MACN 47116	-24.20	-62.02
Cerdocyon thous	M	MZUSP 9686	0.97	-61.34
Cerdocyon thous	Unknown	MZUSP 9687	0.97	-61.34
Cerdocyon thous	M	MACN 16189	-30.58	-59.95
Cerdocyon thous	Unknown	MACN 24264	-25.22	-59.86
Cerdocyon thous	Unknown	MACN 29839	-25.33	-59.68
Cerdocyon thous	Unknown	MACN 336	-28.04	-59.22
Cerdocyon thous	Unknown	MACN 21228	-27.49	-58.82
Cerdocyon thous	Unknown	MACN 20316	-27.58	-58.75
Cerdocyon thous	Unknown	MACN 24267	-32.22	-58.15
Cerdocyon thous	Unknown	MACN 5045	-25.33	-58.10
Cerdocyon thous	Unknown	MACN 5043	-25.33	-58.10
Cerdocyon thous	Unknown	MACN 5040	-25.33	-58.10

Cerdocyon thous	Unknown	MACN 4534	-22.29	-57.94
Cerdocyon thous	F	MNHN 707	-21.72	-57.89
Cerdocyon thous	M	MNHN 706	-21.72	-57.89
Cerdocyon thous	F	MNHN 869	-30.55	-57.86
Cerdocyon thous	M	MZUSP 3372	-19.01	-57.66
Cerdocyon thous	M	MZUSP 3373	-19.01	-57.66
Cerdocyon thous	Unknown	UFSC 3728	-19.21	-57.65
Cerdocyon thous	Unknown	MNHN 255	-33.40	-57.54
Cerdocyon thous	F	MNHN 262	-30.95	-57.52
Cerdocyon thous	F	MNHN 965	-30.29	-57.20
Cerdocyon thous	Unknown	MN 25606	-21.23	-57.13
Cerdocyon thous	Unknown	MN 25607	-21.23	-57.13
Cerdocyon thous	Unknown	MNHN 253	-33.94	-56.75
Cerdocyon thous	Unknown	MZUSP 19847	-16.26	-56.62
Cerdocyon thous	M	MNHN 2645	-30.65	-56.59
Cerdocyon thous	M	MZUSP 3777	-20.24	-56.36
Cerdocyon thous	F	MNHN 1287	-34.52	-56.28
Cerdocyon thous	F	MNHN 260	-31.88	-56.24
Cerdocyon thous	Unknown	MNHN 2540	-34.82	-56.16
Cerdocyon thous	M	MNHN 829	-31.25	-56.15
Cerdocyon thous	M	MNHN 956	-31.25	-56.15
Cerdocyon thous	M	MZUSP 6314	-15.60	-56.09
Cerdocyon thous	M	MZUSP 6315	-15.60	-56.09
Cerdocyon thous	Unknown	MNHN 5478	-34.72	-56.07
Cerdocyon thous	M	MNHN 2420	-31.73	-55.98
Cerdocyon thous	M	MNHN 251	-31.73	-55.98
Cerdocyon thous	Unknown	MNHN 252	-31.73	-55.98
Cerdocyon thous	F	MNHN 258	-31.71	-55.97
Cerdocyon thous	F	MNHN 2538	-31.70	-55.97
Cerdocyon thous	M	MNHN 1119	-31.70	-55.97
Cerdocyon thous	M	MNHN 1140	-31.70	-55.97
Cerdocyon thous	F	MNHN 1254	-32.84	-55.90
Cerdocyon thous	M	MNHN 6260	-34.55	-55.94
Cerdocyon thous	M	MNHN 6262	-34.55	-55.94
Cerdocyon thous	M	MNHN 6273	-34.55	-55.94
Cerdocyon thous	F	MNHN 2649	-33.55	-55.71
Cerdocyon thous	F	MNHN 2651	-33.55	-55.71
Cerdocyon thous	M	MNHN 2650	-33.55	-55.71
Cerdocyon thous	Unknown	MNHN 2680	-33.93	-55.68
Cerdocyon thous	Unknown	MNHN 4716	-30.91	-55.55
Cerdocyon thous	Unknown	MNHN 6437	-30.91	-55.55
Cerdocyon thous	Unknown	MACN 34676	-27.32	-55.53
Cerdocyon thous	M	MNHN 954	-33.80	-55.53

Cerdocyon thous	Unknown	MN 4908	-21.62	-55.17
Cerdocyon thous	F	MNHN 247	-34.88	-54.97
Cerdocyon thous	M	MNHN 2646	-34.88	-54.97
Cerdocyon thous	F	MNHN 3198	-34.00	-54.96
Cerdocyon thous	Unknown	MNHN 2695	-33.53	-54.94
Cerdocyon thous	Unknown	MNHN 7111	-34.80	-54.91
Cerdocyon thous	Unknown	MNHN 7352	-34.80	-54.91
Cerdocyon thous	M	MACN 20456	-25.66	-54.50
Cerdocyon thous	F	MACN 25802	-25.66	-54.50
Cerdocyon thous	M	MACN 20454	-25.66	-54.50
Cerdocyon thous	F	MACN 20455	-25.66	-54.50
Cerdocyon thous	Unknown	MNHN 249	-27.00	-54.44
Cerdocyon thous	F	MHNCI 3826	-25.68	-54.43
Cerdocyon thous	M	MHNCI 3850	-25.68	-54.43
Cerdocyon thous	Unknown	MHNCI 3811	-25.68	-54.43
Cerdocyon thous	F	MNHN 2643	-33.23	-54.36
Cerdocyon thous	M	MHNCI 489	-24.10	-54.27
Cerdocyon thous	M	MACN 13051	-25.58	-54.07
Cerdocyon thous	Unknown	MZUSP 19738	-19.54	-54.05
Cerdocyon thous	F	MNHN 1055	-29.68	-53.81
Cerdocyon thous	Unknown	MNHN 6410	-34.40	-53.79
Cerdocyon thous	Unknown	MCNFZB 423	-28.64	-53.61
Cerdocyon thous	Unknown	MZUSP 3182	-28.29	-53.49
Cerdocyon thous	Unknown	MZUSP 7149	-28.29	-53.49
Cerdocyon thous	F	MHNCI 164	-22.96	-53.30
Cerdocyon thous	M	MHNCI 60	-22.72	-53.17
Cerdocyon thous	F	MHNCI 3945	-25.20	-53.15
Cerdocyon thous	Unknown	MN 25005	-18.54	-53.12
Cerdocyon thous	F	MHNCI 5235	-22.63	-52.88
Cerdocyon thous	Unknown	MHNCI 5786	-22.63	-52.88
Cerdocyon thous	Unknown	MN 25683	-14.67	-52.35
Cerdocyon thous	M	MN 71092	-14.67	-52.35
Cerdocyon thous	Unknown	MHNCI 3948	-25.84	-52.03
Cerdocyon thous	M	MZUSP 514	-31.37	-51.97
Cerdocyon thous	M	MZUSP 518	-31.37	-51.97
Cerdocyon thous	Unknown	MZUSP 415	-31.37	-51.97
Cerdocyon thous	Unknown	MZUSP 34772	-19.12	-51.74
Cerdocyon thous	Unknown	MZUSP 34775	-19.12	-51.74
Cerdocyon thous	Unknown	MZUSP 34776	-19.12	-51.74
Cerdocyon thous	M	MZUSP 19741	-20.87	-51.61
Cerdocyon thous	M	MZUSP 1935	-20.65	-51.50
Cerdocyon thous	M	MCNFZB 598	-29.69	-51.47
Cerdocyon thous	F	MHNCI 1077	-25.39	-51.46

Cerdocyon thous	F	MZUSP 3762	-21.50	-51.32
Cerdocyon thous	F	MZUSP 3763	-21.50	-51.32
Cerdocyon thous	M	MCP 178	-30.29	-51.30
Cerdocyon thous	M	UFSC 3791	-27.40	-51.23
Cerdocyon thous	F	MHNCI 3972	-26.23	-50.93
Cerdocyon thous	Unknown	MZUSP 6640	-17.79	-50.92
Cerdocyon thous	F	MHNCI 2723	-24.32	-50.62
Cerdocyon thous	Unknown	MN 68361	-24.32	-50.62
Cerdocyon thous	Unknown	UFSC 762	-26.12	-50.31
Cerdocyon thous	Unknown	MHNCI 3923	-25.10	-50.16
Cerdocyon thous	M	MHNCI 1708	-23.30	-50.07
Cerdocyon thous	M	MHNCI 1714	-23.30	-50.07
Cerdocyon thous	M	MHNCI 132	-24.79	-50.01
Cerdocyon thous	M	MHNCI 307	-24.79	-50.01
Cerdocyon thous	M	MHNCI 309	-24.79	-50.01
Cerdocyon thous	M	MHNCI 5706	-25.44	-49.97
Cerdocyon thous	F	MZUSP 2832	-21.46	-49.95
Cerdocyon thous	F	MZUSP 2833	-21.46	-49.95
Cerdocyon thous	F	MZUSP 2835	-21.46	-49.95
Cerdocyon thous	M	MZUSP 2834	-21.46	-49.95
Cerdocyon thous	M	MZUSP 4135	-21.46	-49.95
Cerdocyon thous	M	MHNCI 1717	-24.21	-49.78
Cerdocyon thous	F	MHNCI 1713	-25.46	-49.53
Cerdocyon thous	M	MHNCI 3925	-25.57	-49.39
Cerdocyon thous	F	MZUSP 1164	-24.10	-49.34
Cerdocyon thous	M	MZUSP 1165	-24.10	-49.34
Cerdocyon thous	M	MHNCI 3401	-25.50	-49.34
Cerdocyon thous	M	MHNCI 3728	-25.99	-49.34
Cerdocyon thous	F	MHNCI 6184	-25.51	-49.20
Cerdocyon thous	F	MHNCI 6198	-25.51	-49.20
Cerdocyon thous	M	MHNCI 3941	-25.35	-49.16
Cerdocyon thous	F	UFSC 576	-27.90	-49.13
Cerdocyon thous	M	MHNCI 4043	-25.45	-49.07
Cerdocyon thous	F	MZUSP 486	-22.32	-49.07
Cerdocyon thous	M	MZUSP 463	-22.32	-49.07
Cerdocyon thous	M	MZUSP 487	-22.32	-49.07
Cerdocyon thous	F	UFSC 348	-27.57	-48.98
Cerdocyon thous	F	MN 4891	-16.33	-48.96
Cerdocyon thous	Unknown	MN 25612	-16.33	-48.96
Cerdocyon thous	F	UFSC 297	-27.96	-48.68
Cerdocyon thous	M	MHNCI 252	-25.85	-48.64
Cerdocyon thous	F	MHNCI 54	-25.82	-48.54
Cerdocyon thous	M	MHNCI 56	-25.82	-48.54

Cerdocyon thous	M	MHNCI 57	-25.82	-48.54
Cerdocyon thous	F	UFSC 773	-27.48	-48.53
Cerdocyon thous	F	UFSC 298	-27.56	-48.49
Cerdocyon thous	M	MN 75086	-7.12	-48.45
Cerdocyon thous	M	UFSC 850	-27.44	-48.47
Cerdocyon thous	Unknown	MN 75090	-7.12	-48.45
Cerdocyon thous	F	MZUSP 4221	-13.51	-48.36
Cerdocyon thous	M	MZUSP 19742	-13.51	-48.36
Cerdocyon thous	M	MZUSP 4215	-13.51	-48.36
Cerdocyon thous	M	MZUSP 4216	-13.51	-48.36
Cerdocyon thous	M	MZUSP 4219	-13.51	-48.36
Cerdocyon thous	F	MZUSP 13797	-23.02	-48.01
Cerdocyon thous	F	MZUSP 2919	-20.55	-47.41
Cerdocyon thous	M	MZUSP 2917	-20.55	-47.41
Cerdocyon thous	M	MZUSP 2918	-20.55	-47.41
Cerdocyon thous	F	MN 25589	-20.72	-46.61
Cerdocyon thous	F	MN 25590	-20.72	-46.61
Cerdocyon thous	M	MN 25591	-20.72	-46.61
Cerdocyon thous	M	MZUSP 6636	-23.29	-46.59
Cerdocyon thous	M	MZUSP 4217	-13.39	-46.33
Cerdocyon thous	F	MN 5505	-23.44	-45.08
Cerdocyon thous	F	MZUSP 3034	-17.34	-44.92
Cerdocyon thous	F	MZUSP 3039	-17.34	-44.92
Cerdocyon thous	F	MZUSP 3042	-17.34	-44.92
Cerdocyon thous	F	MZUSP 3098	-17.34	-44.92
Cerdocyon thous	F	MZUSP 3104	-17.34	-44.92
Cerdocyon thous	M	MZUSP 3031	-17.34	-44.92
Cerdocyon thous	M	MZUSP 3032	-17.34	-44.92
Cerdocyon thous	M	MZUSP 3033	-17.34	-44.92
Cerdocyon thous	M	MZUSP 3043	-17.34	-44.92
Cerdocyon thous	M	MZUSP 3101	-17.34	-44.92
Cerdocyon thous	F	MN 42800	-22.50	-44.56
Cerdocyon thous	M	MZUSP 4220	-15.46	-44.32
Cerdocyon thous	F	MN 3885	-22.96	-44.04
Cerdocyon thous	M	MN 3887	-22.96	-44.04
Cerdocyon thous	M	MZUSP 3038	-17.75	-44.37
Cerdocyon thous	Unknown	MN 3036	-19.28	-44.41
Cerdocyon thous	M	MN 29061	-14.76	-43.93
Cerdocyon thous	M	MN 29062	-14.76	-43.93
Cerdocyon thous	F	MN 4242	-19.63	-43.89
Cerdocyon thous	M	MN 4243	-19.63	-43.89
Cerdocyon thous	Unknown	MN 46810	-15.48	-43.60
Cerdocyon thous	Unknown	MN 63517	-9.01	-42.70

Cerdocyon thous	Unknown	MN 63491	-8.84	-42.50
Cerdocyon thous	Unknown	MN 25594	-22.66	-42.38
Cerdocyon thous	Unknown	MN 75981	-8.33	-42.31
Cerdocyon thous	Unknown	MN 63471	-8.36	-42.22
Cerdocyon thous	Unknown	MN 43968	-22.96	-42.03
Cerdocyon thous	Unknown	MN 72784	-22.37	-41.78
Cerdocyon thous	Unknown	MN 25578	-4.05	-40.87
Cerdocyon thous	F	MN 25597	-19.93	-40.60
Cerdocyon thous	F	MZUSP 2595	-10.47	-40.18
Cerdocyon thous	F	MZUSP 2598	-10.47	-40.18
Cerdocyon thous	F	MZUSP 2599	-10.47	-40.18
Cerdocyon thous	F	MZUSP 2602	-10.47	-40.18
Cerdocyon thous	M	MZUSP 19757	-10.47	-40.18
Cerdocyon thous	M	MZUSP 2594	-10.47	-40.18
Cerdocyon thous	M	MZUSP 2596	-10.47	-40.18
Cerdocyon thous	M	MZUSP 2600	-10.47	-40.18
Cerdocyon thous	M	MZUSP 2601	-10.47	-40.18
Cerdocyon thous	Unknown	MN 32370	-15.60	-39.86
Cerdocyon thous	M	MZUSP 3504	-14.79	-39.28
Cerdocyon thous	F	MN 10987	-14.80	-39.03
Cerdocyon thous	F	MN 11205	-14.80	-39.03
Cerdocyon thous	M	MN 25577	-4.33	-38.88
Cerdocyon thous	M	MZUSP 8732	-4.33	-38.88
Cerdocyon thous	Unknown	MN 25570	-4.33	-38.88
Cerdocyon thous	Unknown	MZUSP 3831	-12.95	-38.50
Cerdocyon thous	F	MN 25584	-8.89	-36.50
Cerdocyon thous	F	MN 25608	-8.89	-36.50
Cerdocyon thous	M	MN 25583	-8.89	-36.50
Chrysocyon brachyurus	Unknown	MACN 24043	-28.28	-57.19
Chrysocyon brachyurus	Unknown	MACN 25103	-28.87	-56.33
Chrysocyon brachyurus	Unknown	MACN 24201	-28.87	-56.33
Chrysocyon brachyurus	M	MN 71086	-16.19	-55.94
Chrysocyon brachyurus	M	MN 70999	-14.57	-52.45
Chrysocyon brachyurus	Femea	MPEG 1419	-15.90	-52.24
Chrysocyon brachyurus	M	UFSC 356	-28.63	-50.43
Chrysocyon brachyurus	Unknown	MHNCI 4250	-24.57	-50.27
Chrysocyon brachyurus	M	MHNCI 6087	-25.09	-50.16
Chrysocyon brachyurus	Femea	MHNCI 3522	-25.42	-50.01
Chrysocyon brachyurus	Femea	MHNCI 4246	-25.42	-50.01
Chrysocyon brachyurus	M	MHNCI 4244	-25.42	-50.01
Chrysocyon brachyurus	Unknown	MHNCI 5080	-25.42	-50.01
Chrysocyon brachyurus	F	MZUSP 32042	-22.88	-49.24
Chrysocyon brachyurus	M	MZUSP 32039	-22.88	-49.24

Chrysocyon brachyurus	Unknown	MN 36666	-13.53	-48.21
Chrysocyon brachyurus	Unknown	MHNCI 2699	-21.14	-47.99
Chrysocyon brachyurus	Unknown	MZUSP 3700	-20.54	-47.40
Chrysocyon brachyurus	Unknown	MZUSP 31981	-18.58	-47.87
Chrysocyon brachyurus	F	MZUSP 3025	-22.37	-46.94
Chrysocyon brachyurus	Unknown	UFSC 372	-16.37	-46.85
Chrysocyon brachyurus	M	MN 17501	-20.72	-46.61
Chrysocyon brachyurus	Unknown	MN 1049	-19.73	-44.67
Chrysocyon brachyurus	Unknown	MN 640	-19.73	-44.67
Chrysocyon brachyurus	Unknown	MN 17502	-20.33	-43.56
Lycalopex culpaeus	F	MACN 3159	-0.48	-78.14
Lycalopex culpaeus	M	MACN 15109	-39.75	-70.61
Lycalopex culpaeus	F	MACN 15088	-39.75	-70.61
Lycalopex culpaeus	M	MACN 15102	-39.75	-70.61
Lycalopex culpaeus	M	MACN 15184	-39.75	-70.61
Lycalopex culpaeus	F	MACN 15190	-39.75	-70.61
Lycalopex culpaeus	F	MACN 15183	-39.75	-70.61
Lycalopex culpaeus	Unknown	MACN 19221	-49.21	-68.19
Lycalopex culpaeus	Unknown	MACN 19222	-49.21	-68.19
Lycalopex culpaeus	F	MACN 24210	-43.67	-66.67
Lycalopex culpaeus	M	MACN 3839	-22.66	-66.24
Lycalopex culpaeus	Unknown	MACN 3069	-27.82	-65.50
Lycalopex culpaeus	Unknown	MACN 4155	-22.216667	-65.23
Lycalopex griseus	Unknown	MACN 16322	-47.87	-72.25
Lycalopex griseus	Unknown	MACN 16321	-47.87	-72.25
Lycalopex griseus	Unknown	MACN 15692	-47.87	-72.25
Lycalopex griseus	F	MACN 20208	-45.92	-71.64
Lycalopex griseus	Unknown	MACN 2087	-51.46	-70.83
Lycalopex griseus	F	MACN 20205	-51.46	-70.83
Lycalopex griseus	F	MACN 20206	-51.46	-70.83
Lycalopex griseus	Unknown	MACN 225	-45.04	-70.82
Lycalopex griseus	Unknown	MACN 224	-45.04	-70.82
Lycalopex griseus	M	MACN 20207	-41.13	-70.72
Lycalopex griseus	F	MACN 20278	-41.13	-70.72
Lycalopex griseus	F	MACN 15185	-39.75	-70.61
Lycalopex griseus	F	MACN 15264	-39.75	-70.61
Lycalopex griseus	M	MACN 15265	-39.75	-70.61
Lycalopex griseus	M	MACN 15263	-39.75	-70.61
Lycalopex griseus	M	MACN 15262	-39.75	-70.61
Lycalopex griseus	Unknown	MACN 14902	-39.03	-70.40
Lycalopex griseus	Unknown	MACN 25364	-34.87	-69.60
Lycalopex griseus	Unknown	MACN 20206	-46.67	-68.93
Lycalopex griseus	Unknown	MACN 363	-49.21	-68.19

Lycalopex griseus	F	MACN 13781	-30.12	-68.00
Lycalopex griseus	Unknown	MACN 20829	-34.05	-67.97
Lycalopex griseus	Unknown	MACN 24263	-54.49	-67.67
Lycalopex griseus	Unknown	MACN 2453	-39.03	-67.58
Lycalopex griseus	Unknown	MACN 2452	-39.03	-67.58
Lycalopex griseus	Unknown	MACN 2479	-39.03	-67.58
Lycalopex griseus	Unknown	MACN 25367	-26.11	-67.43
Lycalopex griseus	F	MACN 50420	-27.93	-67.19
Lycalopex griseus	Unknown	MACN 51170	-28.29	-66.34
Lycalopex griseus	Unknown	MACN 50432	-27.60	-66.32
Lycalopex griseus	F	MACN 50419	-27.60	-66.32
Lycalopex griseus	Unknown	MACN 16325	-43.80	-65.75
Lycalopex griseus	Unknown	MACN 2333	-31.66	-64.43
Lycalopex gymnocercus	F	MHNCI 231	-25.42	-50.00
Lycalopex gymnocercus	Unknown	MACN 23290	-31.64	-60.68
Lycalopex gymnocercus	M	MACN 34317	-29.39	-66.98
Lycalopex gymnocercus	F	MACN 50433	-27.60	-66.32
Lycalopex gymnocercus	F	MACN 532	-27.86	-65.95
Lycalopex gymnocercus	F	MACN 15992	-36.57	-65.67
Lycalopex gymnocercus	F	MACN 15938	-36.57	-65.67
Lycalopex gymnocercus	M	MACN 15932	-36.57	-65.67
Lycalopex gymnocercus	M	MACN 15958	-36.57	-65.67
Lycalopex gymnocercus	M	MACN 15966	-36.57	-65.67
Lycalopex gymnocercus	F	MACN 16063	-36.57	-65.67
Lycalopex gymnocercus	M	MACN 15601	-37.99	-65.59
Lycalopex gymnocercus	F	MACN 28182	-27.16	-65.50
Lycalopex gymnocercus	Unknown	MACN 24266	-42.39	-65.17
Lycalopex gymnocercus	Unknown	MACN 2935	-32.08	-65.14
Lycalopex gymnocercus	F	MACN 14323	-25.49	-64.97
Lycalopex gymnocercus	F	MACN 14319	-25.49	-64.97
Lycalopex gymnocercus	F	MACN 30150	-26.59	-64.81
Lycalopex gymnocercus	F	MACN 39194	-30.72	-64.81
Lycalopex gymnocercus	F	MACN 50498	-37.38	-64.60
Lycalopex gymnocercus	M	MACN 50495	-37.38	-64.60
Lycalopex gymnocercus	F	MACN 50491	-37.38	-64.60
Lycalopex gymnocercus	M	MACN 50502	-37.38	-64.60
Lycalopex gymnocercus	F	MACN 50505	-37.38	-64.60
Lycalopex gymnocercus	M	MACN 50494	-37.38	-64.60
Lycalopex gymnocercus	Unknown	MACN 32263	-23.14	-64.32
Lycalopex gymnocercus	M	MACN 24472	-31.40	-64.18
Lycalopex gymnocercus	M	MACN 13313	-29.76	-64.06
Lycalopex gymnocercus	F	MACN 13299	-29.76	-64.06
Lycalopex gymnocercus	F	MACN 49134	-38.69	-63.77

Lycalopex gymnocercus	F	MACN 49160	-38.69	-63.77
Lycalopex gymnocercus	F	MACN 49149	-38.69	-63.77
Lycalopex gymnocercus	M	MACN 49139	-38.69	-63.77
Lycalopex gymnocercus	M	MACN 49148	-38.69	-63.77
Lycalopex gymnocercus	M	MACN 49167	-38.69	-63.77
Lycalopex gymnocercus	Unknown	MNHN 712	-34.01	-62.24
Lycalopex gymnocercus	M	MACN 15389	-37.46	-61.93
Lycalopex gymnocercus	M	MACN 15388	-37.46	-61.93
Lycalopex gymnocercus	F	MACN 15390	-37.46	-61.93
Lycalopex gymnocercus	F	MACN 14386	-34.06	-61.89
Lycalopex gymnocercus	Unknown	MACN 24203	-24.93	-61.48
Lycalopex gymnocercus	Unknown	MNHN 1144	-35.29	-61.42
Lycalopex gymnocercus	M	MACN 15364	-36.23	-61.11
Lycalopex gymnocercus	F	MACN 15363	-36.23	-61.11
Lycalopex gymnocercus	Unknown	MZUSP 653	-31.45	-60.93
Lycalopex gymnocercus	Unknown	MZUSP 651	-31.45	-60.93
Lycalopex gymnocercus	F	MACN 33177	-38.38	-60.28
Lycalopex gymnocercus	Unknown	MACN 2628	-36.78	-59.85
Lycalopex gymnocercus	F	MACN 54133	-37.67	-59.81
Lycalopex gymnocercus	Unknown	MACN 24265	-30.76	-59.64
Lycalopex gymnocercus	F	MACN 14409	-38.56	-58.71
Lycalopex gymnocercus	Unknown	MACN 24208	-32.16	-58.40
Lycalopex gymnocercus	Unknown	MNHN 6280	-33.53	-58.28
Lycalopex gymnocercus	Unknown	MACN 24133	-37.85	-58.26
Lycalopex gymnocercus	Unknown	MACN 24134	-37.85	-58.26
Lycalopex gymnocercus	Unknown	MACN 24140	-37.85	-58.26
Lycalopex gymnocercus	M	MNHN 1940	-34.18	-58.10
Lycalopex gymnocercus	Unknown	MNHN 3125	-34.18	-58.10
Lycalopex gymnocercus	Unknown	MACN 2448	-38.17	-58.10
Lycalopex gymnocercus	Unknown	MACN 24171	-35.58	-58.02
Lycalopex gymnocercus	Unknown	MACN 266	-38.16	-57.72
Lycalopex gymnocercus	F	MNHN 1111	-33.40	-57.54
Lycalopex gymnocercus	F	MNHN 263	-33.40	-57.54
Lycalopex gymnocercus	F	MNHN 268	-33.40	-57.54
Lycalopex gymnocercus	F	MNHN 269	-33.40	-57.54
Lycalopex gymnocercus	F	MNHN 270	-33.40	-57.54
Lycalopex gymnocercus	Unknown	MNHN 281	-33.40	-57.54
Lycalopex gymnocercus	Unknown	MNHN 282	-33.40	-57.54
Lycalopex gymnocercus	F	MNHN 278	-33.97	-57.08
Lycalopex gymnocercus	M	MNHN 2648	-33.97	-57.08
Lycalopex gymnocercus	M	MNHN 1014	-34.17	-56.68
Lycalopex gymnocercus	Unknown	MACN 26162	-36.88	-56.68
Lycalopex gymnocercus	Unknown	MNHN 2699	-31.70	-55.97

Lycalopex gymnocercus	M	MNHN 881	-33.99	-55.65
Lycalopex gymnocercus	M	MNHN 950	-33.99	-55.65
Lycalopex gymnocercus	M	MNHN 1939	-32.90	-55.55
Lycalopex gymnocercus	F	MNHN 1091	-33.80	-55.53
Lycalopex gymnocercus	F	MNHN 713	-33.80	-55.53
Lycalopex gymnocercus	F	MNHN 966	-33.80	-55.53
Lycalopex gymnocercus	Unknown	MNHN 1071	-33.80	-55.53
Lycalopex gymnocercus	Unknown	MNHN 1082	-33.80	-55.53
Lycalopex gymnocercus	Unknown	MNHN 1217	-33.80	-55.53
Lycalopex gymnocercus	Unknown	MNHN 1218	-33.80	-55.53
Lycalopex gymnocercus	Unknown	MNHN 1219	-33.80	-55.53
Lycalopex gymnocercus	M	MCNFZB 488	-28.17	-55.45
Lycalopex gymnocercus	F	UFSC 4592	-26.78	-52.06
Lycalopex gymnocercus	F	MHNCI 59	-26.48	-51.99
Lycalopex gymnocercus	M	MHNCI 61	-26.48	-51.99
Lycalopex gymnocercus	M	MHNCI 3020	-25.69	-51.65
Lycalopex gymnocercus	Unknown	MCP 168	-30.26	-50.51
Lycalopex gymnocercus	Unknown	MCP 618	-30.26	-50.51
Lycalopex gymnocercus	Unknown	MN 2355	-28.63	-50.43
Lycalopex gymnocercus	F	MHNCI 532	-25.10	-50.16
Lycalopex gymnocercus	M	MHNCI 55	-24.79	-50.01
Lycalopex gymnocercus	F	MHNCI 5583	-25.42	-50.00
Lycalopex gymnocercus	M	MHNCI 229	-25.42	-50.00
Lycalopex gymnocercus	Unknown	UFSC 329	-28.34	-49.63
Lycalopex gymnocercus	F	MNHN 271	-33.60	-57.90
Lycalopex gymnocercus	M	MNHN 273	-33.60	-57.90
Lycalopex vetulus	F	MN 5151	-21.61	-55.17
Lycalopex vetulus	F	MN 71112	-14.56	-52.45
Lycalopex vetulus	M	MN 71045	-14.56	-52.45
Lycalopex vetulus	Unknown	MN 71047	-14.56	-52.45
Lycalopex vetulus	Unknown	MN 71093	-14.56	-52.45
Lycalopex vetulus	M	MN 4909	-16.33	-48.95
Lycalopex vetulus	F	MZUSP 1018	-20.54	-47.40
Lycalopex vetulus	F	MZUSP 1011	-20.54	-47.40
Lycalopex vetulus	F	MZUSP 825	-20.54	-47.40
Lycalopex vetulus	F	MZUSP 1015	-20.54	-47.40
Lycalopex vetulus	M	MZUSP 1016	-20.54	-47.40
Lycalopex vetulus	M	MZUSP 1084	-20.54	-47.40
Lycalopex vetulus	Unknown	MZUSP 1012	-20.54	-47.40
Lycalopex vetulus	M	MZUSP 3046	-17.34	-44.93
Lycalopex vetulus	Unknown	MN 3037	-19.54	-44.38
Lycalopex vetulus	F	MN 4241	-19.65	-43.89
Speothos venaticus	M	MACN 5067	-17.46	-63.66

Speothos venaticus	Unknown	MACN 33154	-17.87	-63.00
Speothos venaticus	M	MPEG 6535	2.82	-60.68
Speothos venaticus	M	MN 3035	-14.85	-57.76
Speothos venaticus	M	MPEG 1042	-2.45	-54.70
Speothos venaticus	M	MACN 16510	-25.66	-54.50
Speothos venaticus	Femea	MPEG 5615	-2.49	-54.30
Speothos venaticus	Femea	MPEG 8794	-2.49	-54.30
Speothos venaticus	M	MPEG 8793	-2.49	-54.30
Speothos venaticus	M	MPEG 5614	-2.49	-54.30
Speothos venaticus	F	MZUSP 2684	-26.44	-49.35
Speothos venaticus	Unknown	MZUSP 2685	-26.44	-49.35
Speothos venaticus	M	MPEG 1778	-1.46	-48.50
Speothos venaticus	M	MPEG 1780	-1.46	-48.50
Speothos venaticus	M	MN 25668	-15.79	-47.88

South American canid phylogenetic tree generated using the 10K tree project from Arnold et al. (2010). Branch lengths are proportional to million years. Natural log transformed centroid size was also mapped into the tree using squared change parsimony algorithm (Maddison and Maddison 1991) to provide stronger support to the lack of phylogenetic signal in this trait.

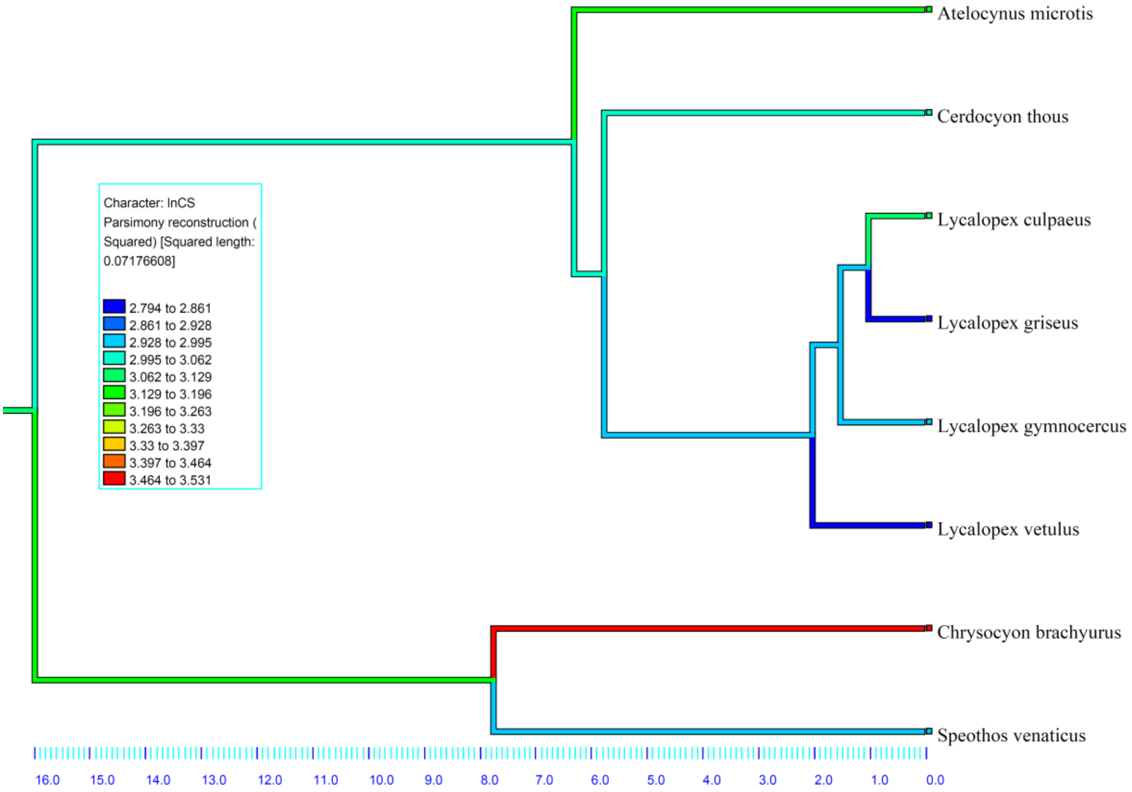

#NEXUS

[created by the 10kTree Website - http://10kTrees.fas.harvard.edu]

BEGIN TREES;

translate

1 Atelocynus_microtis,

2 Chrysocyon_brachyurus,

3 Cerdocyon_thous,

4 Lycalopex_culpaeus,

5 Lycalopex_griseus,

6 Lycalopex_gymnocercus,

7 Lycalopex_vetulus,

8 Speothos_venaticus;

tree consensus_8species = ((1:6.255354,(3:5.718385,(((4:0.981755,5:0.981755):0.484353,6:1.466108):0.515162,7:1.981270):3.737116):0.536969):9.688054,(2:7.698185,8:7.698185):8.245222);

END;

Regression plot showing the effect of size (lnCS) on canid skull shape using 431 specimens. Every species is labelled according to different colour and symbol. Deformation grids show relative shape changes from the smaller (left, lnCS = 2.72) to the larger (right, lnCS = 3.55) individual.

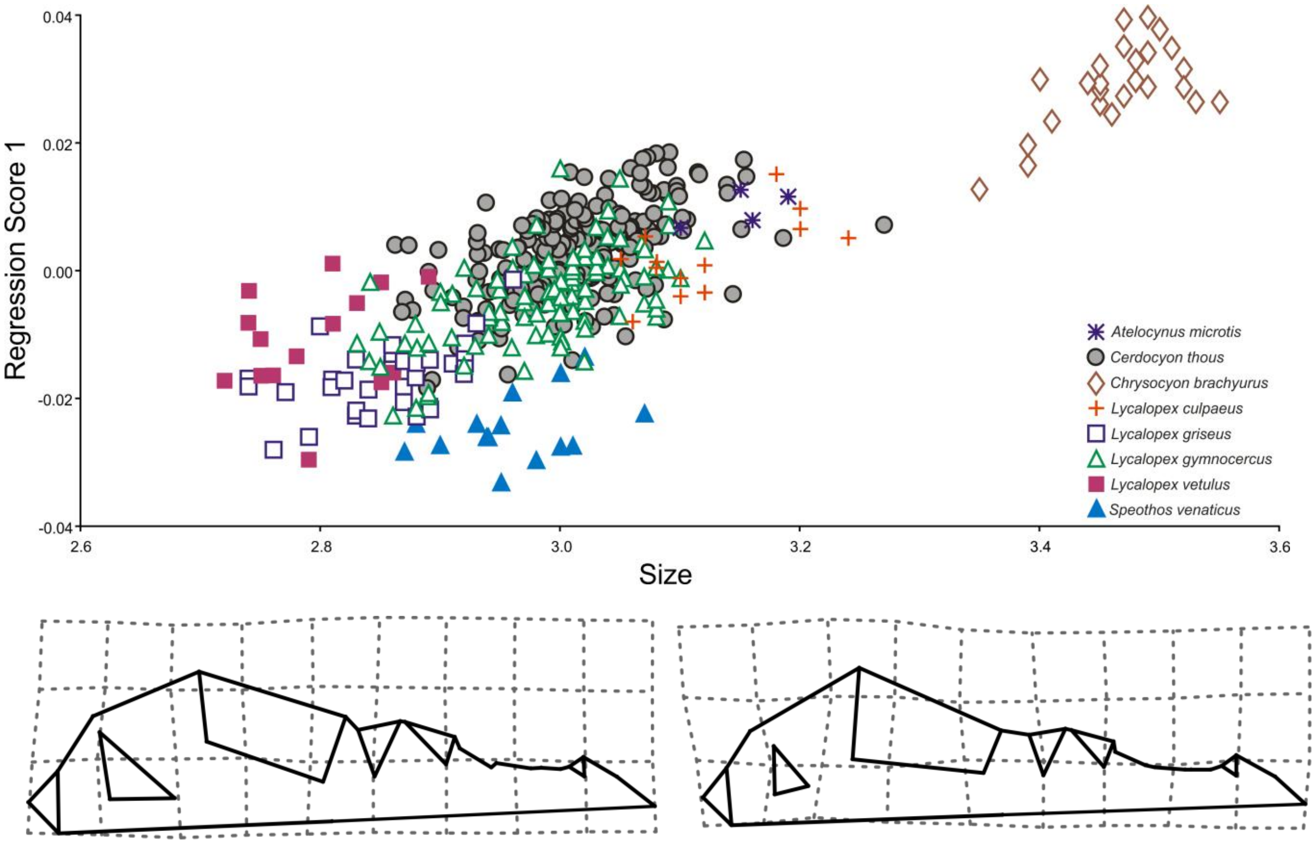

Regression plot showing the effect of size (lnCS) on canid skull shape using 262 locality averaged sub-samples. Every species is labelled according to different colour and symbol. Deformation grids show relative shape changes from the smaller (left, lnCS = 2.74) to the larger (right, lnCS = 3.55) individual.

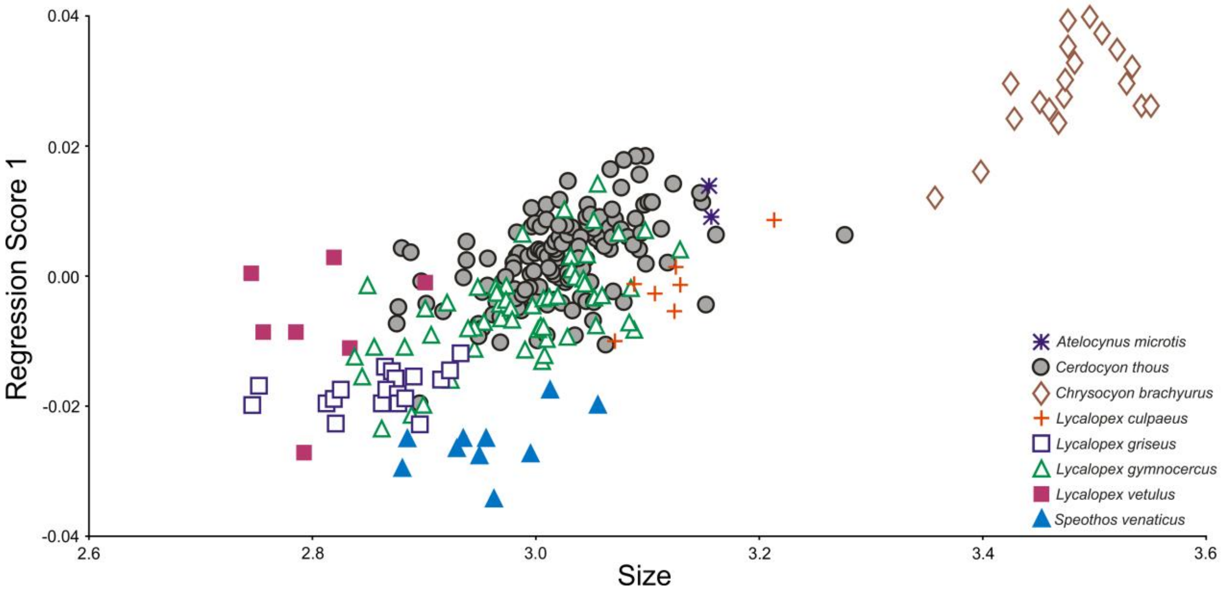

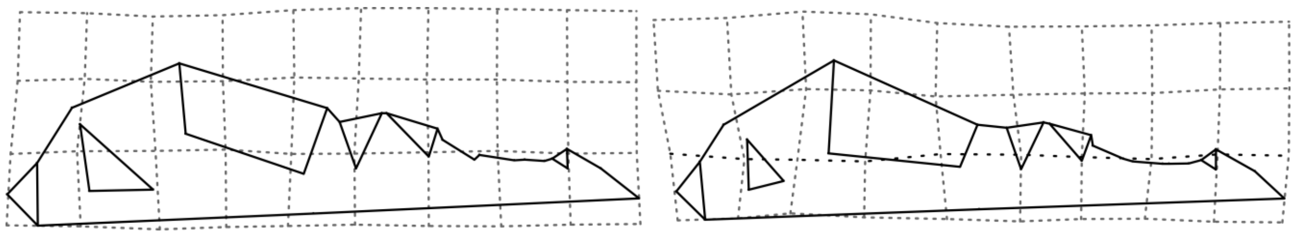

Regression deformation grids showing relative shape changes from the smaller (left, lnCS = 2.86) to the larger (right, lnCS = 3.27) individual of *Cerdocyon thous*.

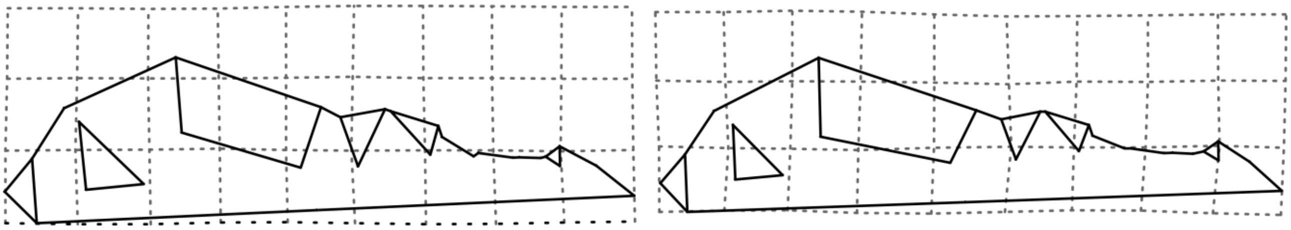

Regression deformation grids showing relative shape changes from the smaller (left, lnCS = 3.35) to the larger (right, lnCS = 3.55) individual of *Chrysocyon brachyurus*.

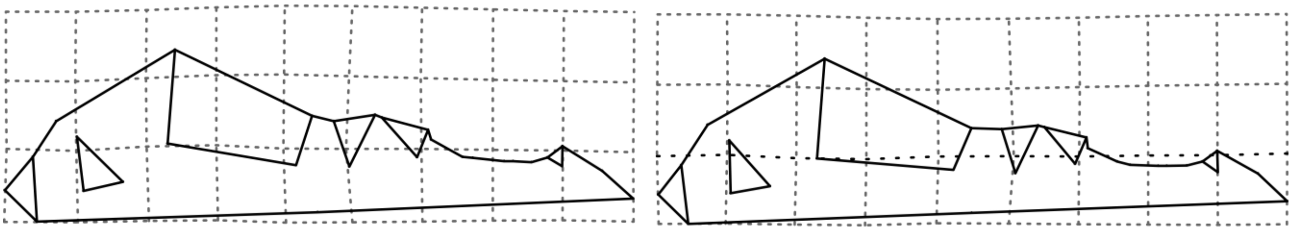

Regression deformation grids showing relative shape changes from the smaller (left, lnCS = 2.83) to the larger (right, lnCS = 3.12) individual of *Lycalopex gymnocercus*.

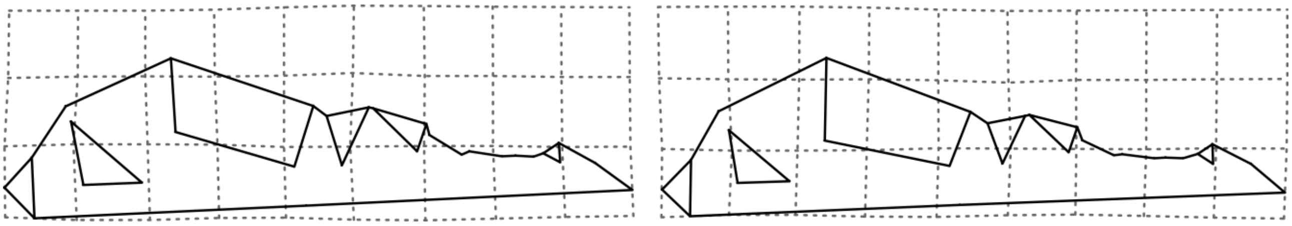

Regression deformation grids showing relative shape changes from the smaller (left, lnCS = 2.72) to the larger (right, lnCS = 3.24) individual of the *Lycalopex* genera.

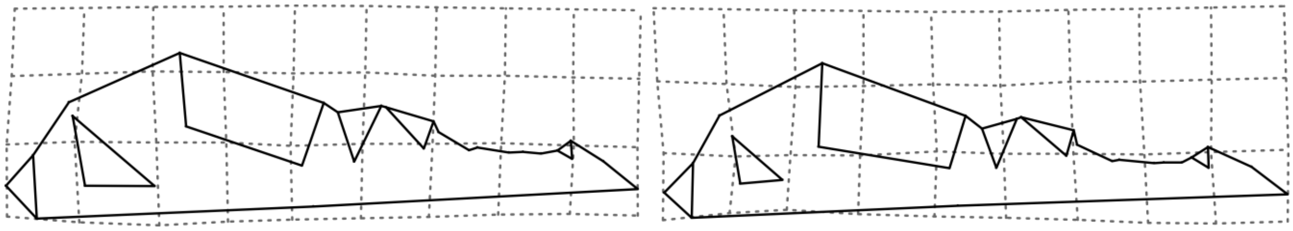

Regression deformation grids showing relative shape changes from the smaller (left, lnCS = 2.87) to the larger (right, lnCS = 3.07) individual of *Speothos venaticus*.

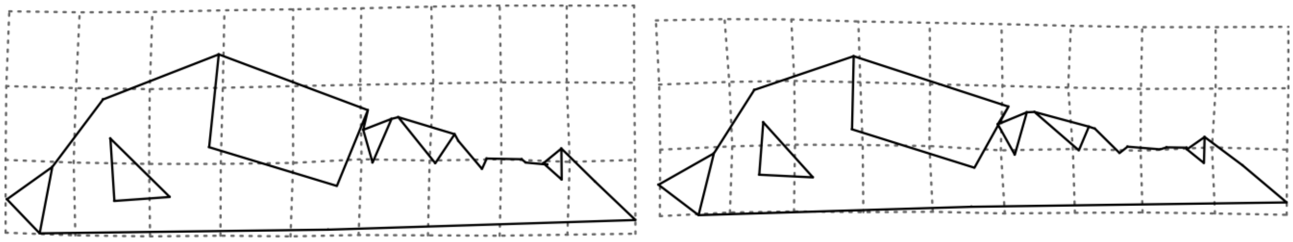

Variation partitioning with skull shape of canids species as dependent variable and taxonomy, size, climate and competition as dependent variables. P values tests for the significance of F after 1000 permutations. Significance is highlighted.

	Df	R^2	Adj. R^2	F	P value
Taxonomy	7	0.49983	0.48604	36.261	**0.005**
Size	1	0.08589	0.08237	24.429	**0.005**
Climate	19	0.26398	0.20619	4.568	**0.005**
Competition	3	0.08386	0.0732	7.872	**0.005**
Taxonomy + Size	8	0.50608	0.49047	32.404	**0.005**
Taxonomy + Climate	26	0.56103	0.51247	11.552	**0.005**
Taxonomy + Competition	10	0.51103	0.49155	26.233	**0.005**
Size + Climate	20	0.3427	0.28815	6.283	**0.005**
Size + Competition	4	0.15128	0.13807	11.452	**0.005**
Competition + Climate	22	0.29931	0.23481	4.640	**0.005**
Taxonomy + Size + Climate	27	0.56575	0.51564	11.291	**0.005**
Taxonomy + Size + Competition	11	0.51608	0.49479	24.238	**0.005**
Taxonomy + Climate + Competition	29	0.57141	0.51783	10.666	**0.005**
Size + Climate + Competition	23	0.36594	0.30467	5.972	**0.005**
All	30	0.57507	0.51988	10.421	**0.005**
Taxonomy “Pure”	7		0.21522	16.241	**0.005**
Size “Pure”	1		0.00205	1.991	**0.035**
Climate “Pure”	19		0.02509	1.688	**0.005**
Competition “Pure”	3		0.00424	1.689	**0.01**

Variation partitioning with skull size of canids species as dependent variable and taxonomy, climate and competition as dependent variables. P values tests for the significance of F after 1000 permutations. Significance is highlighted.

	Df	R^2	Adj. R^2	F	P value
Taxonomy	7	0.84305	0.83873	194.91	**0.001**
Climate	19	0.15318	0.08669	2.3039	**0.005**
Competition	3	0.13405	0.12398	13.312	**0.001**
Taxonomy + Climate	26	0.88041	0.86718	66.54	**0.001**
Taxonomy + Competition	10	0.85391	0.84809	146.72	**0.001**
Competition + Climate	22	0.22998	0.1591	3.2446	**0.001**
All	29	0.89205	0.87856	66.111	**0.001**
Taxonomy “Pure”	7		0.71946	203.28	**0.001**
Climate “Pure”	19		0.03047	4.3141	**0.001**
Competition “Pure”	3		0.01138	8.3417	**0.001**

Schematic depiction of the factors analyzed in partition variation to illustrate both their individual contribution and their interaction components in the variance of shape (A) and size (B) of *Cerdocyon* and shape (C) and size of *Lycalopex* (D). Values < 0 not shown.

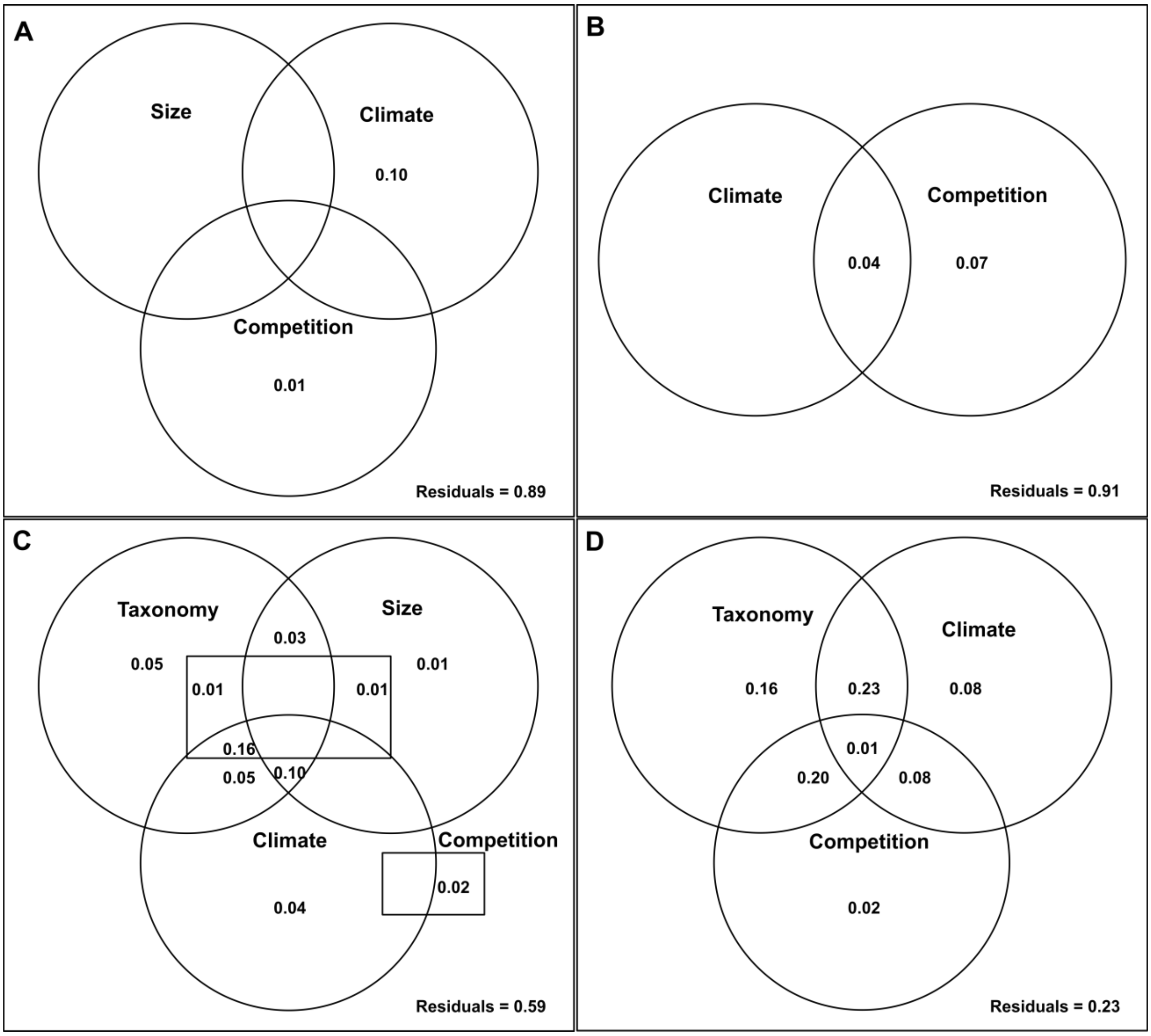

Variation partitioning with skull shape of *Cerdocyon* as dependent variable and size, climate and competition as dependent variables. P values tests for the significance of F after 1000 permutations. Significance is highlighted.

	Df	R^2	Adj. R^2	F	P value
Size	1	0.00973	0.00271	1.3856	0.150
Climate	19	0.22307	0.10306	1.8587	**0.005**
Competition	3	0.03016	0.00923	1.441	**0.045**
Size + Climate	20	0.22962	0.10333	1.8182	**0.005**
Size + Competition	4	0.0378	0.00991	1.3554	0.055
Climate + Competition	22	0.25039	0.11297	1.822	**0.005**
All	23	0.25587	0.11204	1.779	**0.005**
Size "Pure"	1		-0.00092	0.8752	0.555
Climate "Pure"	19		0.10213	1.8354	**0.005**
Competition "Pure"	3		0.00871	1.3989	0.085

Variation partitioning with skull size of *Cerdocyon* as dependent variable and climate and competition as dependent variables. P values tests for the significance of F after 1000 permutations. Significance is highlighted.

	Df	R^2	Adj. R^2	F	P value
Climate	19	0.15196	0.02096	1.16	0.300
Competition	3	0.12683	0.10799	6.7303	**0.005**
All	22	0.22843	0.08698	1.6149	**0.035**
Climate "Pure"	19		-0.02101	0.8316	0.670
Competition "Pure"	3		0.06602	3.9645	**0.020**

Variation partitioning with skull shape of *Lycalopex* as dependent variable and taxonomy, size, climate and competition as dependent variables. P values tests for the significance of F after 1000 permutations. Significance is highlighted.

	Df	R^2	Adj. R^2	F	P value
Taxonomy	3	0.35301	0.32963	15.096	**0.005**
Size	1	0.08423	0.07345	7.8176	**0.005**
Climate	19	0.43322	0.27249	2.6954	**0.005**
Competition	3	0.14819	0.1174	4.8133	**0.005**
Taxonomy + Size	4	0.381	0.35081	12.618	**0.005**
Taxonomy + Climate	22	0.53388	0.37365	3.3321	**0.005**
Taxonomy + Competition	6	0.40021	0.35523	8.8967	**0.005**
Size + Climate	20	0.48304	0.32639	3.0836	**0.005**
Size + Competition	4	0.30464	0.27072	8.9811	**0.005**
Competition + Climate	22	0.48695	0.31058	2.7611	**0.005**
Taxonomy + Size + Climate	23	0.5555	0.39323	3.4233	**0.005**
Taxonomy + Size + Competition	7	0.42343	0.37234	8.2881	**0.005**
Taxonomy + Climate + Competition	25	0.57248	0.39726	3.2674	**0.005**
Size + Climate + Competition	23	0.53043	0.359	3.0942	**0.005**
All	26	0.58954	0.41168	3.3146	**0.005**
Taxonomy “Pure”	3		0.05268	2.8803	**0.005**
Size “Pure”	1		0.01441	2.4944	**0.005**
Climate “Pure”	19		0.03934	1.278	**0.025**
Competition “Pure”	3		0.01845	1.6586	**0.025**

Variation partitioning with skull size of *Lycalopex* as dependent variable and taxonomy, climate and competition as dependent variables. P values tests for the significance of F after 1000 permutations. Significance is highlighted.

	Df	R^2	Adj. R^2	F	P value
Taxonomy	3	0.61023	0.59614	43.316	**0.005**
Climate	19	0.52694	0.39279	3.928	**0.005**
Competition	3	0.32856	0.30429	13.538	**0.005**
Taxonomy + Climate	22	0.81435	0.75053	12.761	**0.005**
Taxonomy + Competition	6	0.71278	0.69124	33.089	**0.005**
Climate + Competition	22	0.71151	0.61234	7.1747	**0.005**
All	25	0.83624	0.76913	12.46	**0.005**
Taxonomy "Pure"	3		0.15679	15.488	**0.005**
Climate "Pure"	19		0.07788	2.4204	**0.01**
Competition "Pure"	3		0.01859	2.7181	0.055
